# Supplementary material for: Associations between adverse working hours and nurses’ sickness absence: a longitudinal analysis of e-roster data from acute hospital wards
Source: BMJ Open. 2026 Jul 21;16(7):e120066. doi: 10.1136/bmjopen-2026-120066 (PMC13410695; doi:10.1136/bmjopen-2026-120066)

SUPPLEMENTARY MATERIAL

Supplementary Table S1. Shift pattern configurations worked by full-time nurses in the previous 7 days

Shift Variables	Overall		2015		2016		2017		2018		2019		2020	
	Mean (SD)	Median	Mean (SD)	Median	Mean (SD)	Median	Mean (SD)	Median	Mean (SD)	Median	Mean (SD)	Median	Mean (SD)	Median
Total hours	34.3 (5.0)	37.5	33.9 (4.8)	37.5	33.9 (4.7)	37.5	34.1 (4.5)	37.5	33.9 (4.9)	37.5	34.6 (5.3)	37.5	35.0 (5.6)	37.5
N shifts	2.9 (0.5)	3.0	2.9 (0.5)	3.0	2.9 (0.5)	3.0	2.9 (0.4)	3.0	2.9 (0.5)	3.0	2.9 (0.5)	3.0	2.9 (0.5)	3.0
Avg shift length	12.0 (1.1)	12.5	12.0 (1.1)	12.5	12.0 (1.1)	12.5	12.0 (1.1)	12.5	12.0 (1.1)	12.5	12.0 (1.0)	12.5	12.1 (1.0)	12.5
N long shifts	2.5 (0.7)	3.0	2.4 (0.8)	3.0	2.4 (0.7)	3.0	2.5 (0.7)	3.0	2.4 (0.7)	3.0	2.5 (0.8)	3.0	2.5 (0.8)	3.0
Prop long shifts	0.9 (0.2)	1.0	0.9 (0.3)	1.0	0.9 (0.2)	1.0	0.9 (0.2)	1.0	0.9 (0.2)	1.0	0.9 (0.2)	1.0	0.9 (0.2)	1.0
N night shifts	1.0 (0.8)	1.0	1.0 (0.7)	1.0	1.0 (0.7)	1.0	1.0 (0.7)	1.0	1.0 (0.8)	1.0	1.0 (0.8)	1.0	1.1 (0.8)	1.0
Prop night shifts	0.3 (0.3)	0.3	0.3 (0.2)	0.3	0.3 (0.2)	0.3	0.3 (0.2)	0.3	0.3 (0.3)	0.3	0.4 (0.3)	0.3	0.4 (0.3)	0.3
N long spells	0.0 (0.0)	0.0	0.0 (0.0)	0.0	0.0 (0.0)	0.0	0.0 (0.0)	0.0	0.0 (0.0)	0.0	0.0 (0.0)	0.0	0.0 (0.0)	0.0
N intense spells	0.1 (0.1)	0.0	0.1 (0.1)	0.0	0.1 (0.1)	0.0	0.1 (0.1)	0.0	0.1 (0.1)	0.0	0.2 (0.2)	0.0	0.2 (0.2)	0.0
N quick returns	0.9 (0.4)	1.0	0.9 (0.4)	1.0	0.9 (0.4)	1.0	0.9 (0.4)	1.0	0.9 (0.4)	1.0	1.0 (0.4)	1.0	1.0 (0.5)	1.0
N short returns	0.2 (0.2)	0.0	0.2 (0.2)	0.0	0.2 (0.2)	0.0	0.2 (0.2)	0.0	0.2 (0.2)	0.0	0.2 (0.2)	0.0	0.2 (0.2)	0.0
N shift rotations	0.7 (0.4)	1.0	0.7 (0.4)	1.0	0.7 (0.4)	1.0	0.7 (0.4)	1.0	0.7 (0.4)	1.0	0.7 (0.4)	1.0	0.7 (0.5)	1.0

Supplementary Table S2. Shift pattern configurations worked in the previous 28 days and odds of sickness

Variables	Univariable Models		Multivariable Model	
	OR (95% CI)	Sig (P)	OR (95% CI)	Sig (P)
Main Shift Variables				
Prop long shifts	1.11 (1.02-1.20)	0.013	(refer to Figure S1)	<0.001
Prop night shifts	0.98 (0.91-1.05)	0.610	(refer to Figure S1)	<0.001
N long spells	0.69 (0.59-0.81)	<0.001	0.98 (0.83-1.16)	0.859
N intense spells	0.95 (0.93-0.97)	<0.001	0.99 (0.96-1.02)	0.482
N quick returns	0.98 (0.97-0.99)	<0.001	1.04 (1.02-1.05)	<0.001
N short returns	0.96 (0.94-0.98)	<0.001	0.97 (0.94-1.00)	0.030
N shift rotations	1.00 (0.99-1.01)	0.929	1.04 (1.02-1.06)	<0.001
Covariates				
Mean weekly hours	0.99 (0.99-0.99)	<0.001	0.99 (0.99-0.99)	<0.001
Total bank hours	0.98 (0.98-0.98)	<0.001	0.98 (0.98-0.98)	<0.001
N sickness episodes	0.68 (0.65-0.71)	<0.001	0.64 (0.61-0.68)	<0.001
Part-time status	1.07 (1.03-1.11)	<0.001	0.99 (0.95-1.03)	0.635

Supplementary Table S3. Nonlinear terms for proportion of long shifts and night shifts in the previous 28 days

Variable	Term	Beta	Sig (P)
Prop long shifts	Linear	-1.636	<0.001
	Quadratic	6.5484	<0.001
	Cubic	-4.748	<0.001
Prop night shifts	Linear	-0.484	<0.001
	Quadratic	0.546	0.001

Supplementary Figure S1. Cubic and quadratic curves for proportion of long shifts and night shifts in the previous 28 days

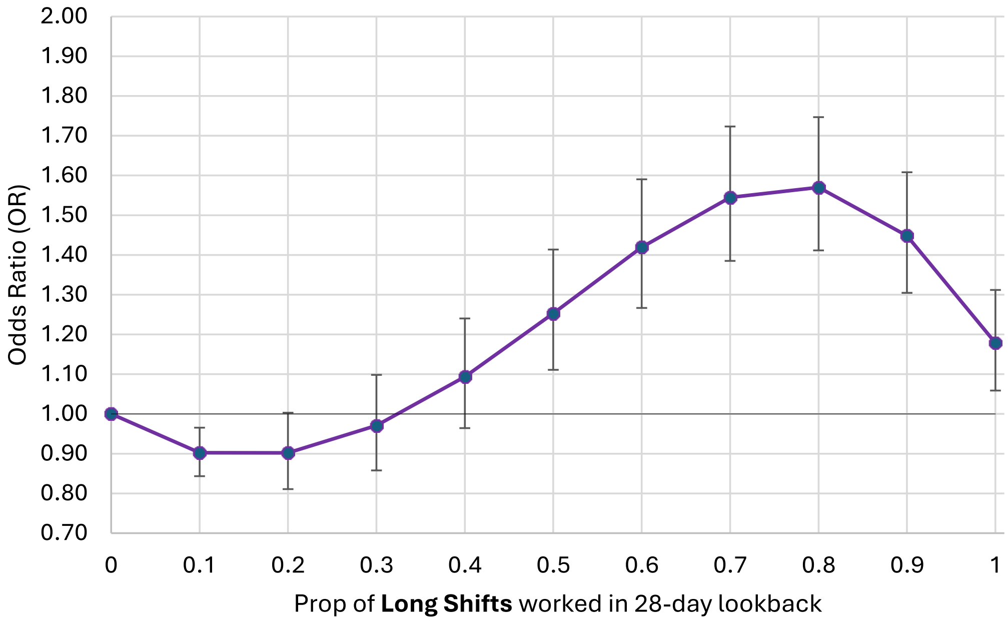

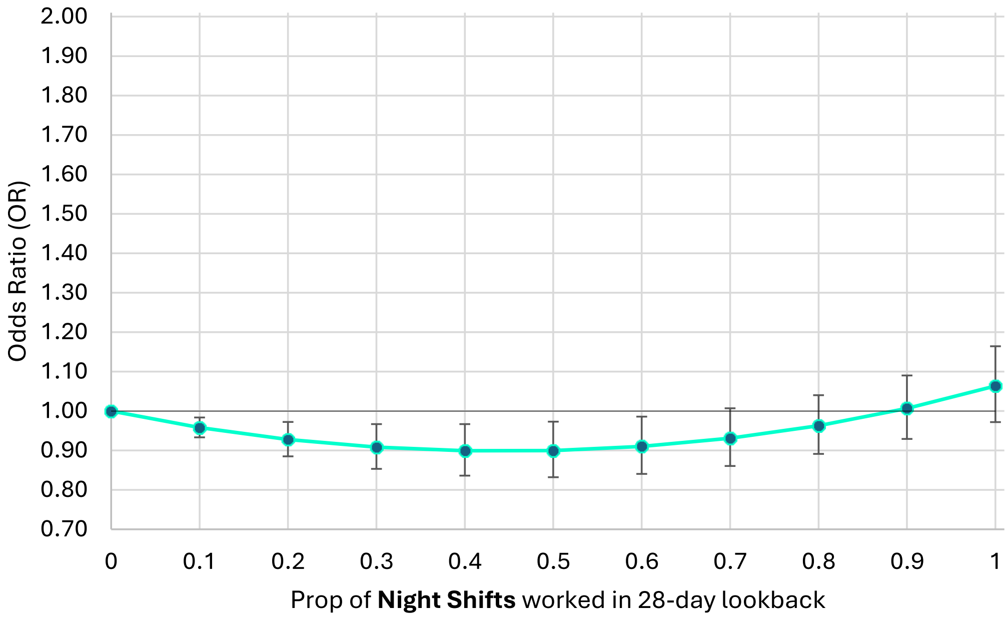

Supplement: Supplementary data [file bmjopen-16-7-s001.pdf]
