# Supplementary material for: Associations between adverse working hours and nurses’ sickness absence: a longitudinal analysis of e-roster data from acute hospital wards
Source: BMJ Open. 2026 Jul 21;16(7):e120066. doi: 10.1136/bmjopen-2026-120066 (PMC13410695; doi:10.1136/bmjopen-2026-120066)
Supplement: Reviewer comments [file bmjopen-2026-120066.reviewer_comments.pdf]

PEER REVIEW HISTORY

BMJ Open publishes all reviews undertaken for accepted manuscripts. Reviewers are asked to complete a checklist review form and are provided with free text boxes to elaborate on their assessment. These free text comments are reproduced below.

ARTICLE DETAILS

Title (Provisional)

Associations between adverse working hours and nurses' sickness absence: a longitudinal analysis of e-roster data from acute hospital wards

Authors

Emmanuel, Talia; Griffiths, Peter; Lamas-Fernandez, Carlos; Dall'Ora, Chiara

---

VERSION 1 - REVIEW

**Reviewer**	**1**
**Name**	**Rahman, Md. Siddikur**
**Affiliation**	**Begum Rokeya University, Statistics**
**Date**	**04-Apr-2026**
**COI**	**None**

The study is valuable and addresses an important workforce and occupational health issue by examining associations between nurses' shift patterns and sickness absence using a large longitudinal dataset. The methodology is appropriate, and the findings are relevant for workforce planning and staff wellbeing. However, several areas require clarification and improvement.

Major Comments:

1. The abstract reports odds ratios but does not clearly define the reference categories for shift patterns. Please specify comparison groups.

2. The rationale for choosing a 7-day exposure window should be presented in the objective or methods section.

3. The term "long shifts" is not defined. Provide the duration (e.g., ≥12 hours).

4. Confounding variables included in the logistic mixed-effects model should be briefly mentioned.

5. The interpretation of the OR for 100% night shifts (OR 1.07) appears small; discuss its practical significance.

Minor Comments:

1. Maintain consistent formatting of confidence intervals (e.g., 95% CI 1.19–1.27).

2. Replace “higher odds for the start of a sickness episode” with “higher odds of sickness absence.”

3. Consider shortening the results section for clarity.

Reviewer 2

Name Yadav, Bijesh

Affiliation King Abdullah International Medical Research Center, Division of Biostatistics, Department of Population Health

Date 13-Apr-2026

COI None

Statistical analysis was not done properly. It is recommended to meet a proper statistician for statistical analysis of this paper.

Reviewer 3

Name Dack, Kyle

Affiliation University of Bristol, MRC Integrative Epidemiology Unit

Date 03-May-2026

COI None

Statistical review summary:

The manuscript is methodologically strong, and the design/analysis/interpretation are all aligned well. Some elements of the modelling strategy are slightly unclear, and would benefit from more information and rationale. Repeated measures from each nurse were accounted for in the mixed-effect model, but something not addressed in the paper is whether other sources of clustering are possible - e.g. there are 95 wards, could there be systematic variation in sickness and shift patterns between wards?

Abstract:

1. The abstract is very clear but two details could be added: The study dates, and the effective N (7,515 nurses in 95 wards).

2. "had the higher odds for sickness (OR 1.68 and 1.07 respectively, p<0.001)" I suggest rephrasing to make this clearer, for example, "were associated with increased odds of sickness".

Methods:

3. The methods would generally be clearer if STROBE-like headings were used, e.g. study design, setting, participants, variables, so that readers can easily locate information.

4. Repeated measures appear to be handled by including a random intercept of nurse ID, but this is not directly stated in the methods. The description is slightly ambiguous, with separate mentions of a random intercept and random effect. It would be helpful to directly define the specification of the mixed effects model.

The ICC justification is somewhat unnecessary, because the data contains non-independent repeated measures which would violate the assumptions of standard regression models, so this data requires a mixed model regardless of ICC.

5. To help prepare readers and align with STROBE I recommend adding a full specification of the multivariable model: the outcome, exposure, and covariates. There is mention of control variables but listing these and their purpose (to minimize confounding or improve precision?), would ensure this is fully transparent.

6. " Multivariable models with the smallest Akaike information criterion (AIC) and Bayesian information criterion (BIC) values were selected for interpretation." It sounds as if most elements of the model are fixed based on prior theory. Which aspects were allowed to vary and were optimized based on AIC/BIC?

Results:

7. The nurses were from 95 different wards. It is possible that there could be variation in both shift patterns and sickness by ward due to differences in practice, which could introduce ward-level clustering. This could be explored descriptively and if there is evidence of clustering, accounting for it with another random intercept or as a fixed effect (covariate adjustment). With so many wards this may not be feasible (e.g. low power, poor model convergence) but it would be useful to explore and discuss - even if it is just to confirm there is no evidence of clustering.

8. Tables: The tables could be improved by adding the N, as some may differ from the main N - e.g. how many are full-time/part-time, how many had adequate data for multivariable modelling.

9. Table 2. The table instructs readers to refer to figure 1 for the multivariable results of the proportional results, but this figure does not show the uncertainty which makes it difficult to interpret.

10. Table 2. Please add specific p-values where they are not too small, as this is recommended in STROBE.

11. Collinearity is not commented on in the results, but the model in table 2 contains a lot of exposures or covariates that overlap conceptually or possibly even mathematically. Almost all were found to be significant which perhaps indicates this is not a major issue. But if there are exposures which overlap substantially, or even occur downstream from one another, this

could split the effect of interest, which would bias the odds ratio towards the null. The results could comment on collinearity results in the methods, and/or add rationale to the methods for modelling all exposures together.

Discussion

12. The interpretation is reasonable but there is occasional causal language (negative effects for example) which go beyond what the data can demonstrate - the data was observational and with limited individual-level data to minimize potential confounding effects.

Otherwise the discussion is strong, and my only minor suggestion is to comment on whether or not ward-based clustering was plausible and would impact the interpretation.

VERSION 1 - AUTHOR RESPONSE

**Reviewer 1**		
Comment	Authors' Response	Page
The abstract reports odds ratios but does not clearly define the reference categories for shift patterns. Please specify comparison groups.	We thank the reviewer for this comment and have revised the abstract to explicitly state comparison groups for each type of shift work exposure variable (counts/frequencies, proportions): "Higher odds of the start of a sickness episode were significantly associated with **each additional rest period of fewer than 11 hours** (OR 1.23, 95% CI 1.19-1.27, p<0.001), **change between day and night shifts** (OR 1.09, 95%CI 1.04-1.13, p<0.001), and **spell of 3 or more consecutive shifts** (OR 1.24, 95% CI 1.16-1.32, p<0.001) in the previous 7 days. By accounting for nonlinearity, analyses revealed that working >70% of shifts as long shifts and 100% of shifts as night shifts had the highest odds of sickness (OR 1.68 and 1.07 respectively, p<0.001) **when compared with working none of these shift types**."	1
The rationale for choosing a 7-day exposure window should be presented in the objective or methods section.	We have now added text to the Methods section explaining why the 7-day window was selected: "Each variable was defined by a 7-day exposure period, i.e. the measurements taken in rolling windows of 7 days prior to each worked shift and sickness absence episode. **This exposure length reflects the weekly structure through which legal and contractual working-time limits are typically defined**."	3
The term "long shifts" is not defined. Provide the duration (e.g., ≥12 hours).	Thank you for this comment. Long shifts are presently defined in the Introduction and Methods sections of the paper; we agree that including the definition in the Abstract improves clarity: "...analyses revealed that working more than 70% of shifts **as ≥12-hour shifts** and 100% of shifts as night shifts ..."	1

Confounding variables included in the logistic mixed-effects model should be briefly mentioned.	Thank you for this comment. We agree that the covariates included in the logistic mixed-effects models should also be briefly described in the Abstract. We have therefore added the following: "**Logistic mixed-effects models were adjusted for total working hours, bank hours, previous sickness, and part-time status**."	1
The interpretation of the OR for 100% night shifts (OR 1.07) appears small; discuss its practical significance.	Thank you for this comment. We agree that the practical significance of this finding requires further interpretation and have amended the Discussion section to include the following context: "**While the magnitude of this association was small (OR 1.07), small increases in individual sickness risk may still have operational relevance when concentrated night work occurs repeatedly across large nursing workforces. As an illustrative example, exposure to 100% night shifts was common within our study population, accounting for approximately 16% of all exposure windows (of which 2,845 were followed by sickness absence). This may correspond to roughly 186 additional sickness episodes, or 744 days lost to sickness absence based on the median episode duration of 4 days.**"	8
Maintain consistent formatting of confidence intervals (e.g., 95% CI 1.19–1.27).	Thank you for flagging this formatting issue, we have reviewed the full paper to ensure OR and CIs are reported consistently.	n/a
Replace "higher odds for the start of a sickness episode" with "higher odds of sickness absence."	We have changed the abstract to reflect this more concise phrasing, thank you.	1
Consider shortening the results section for clarity.	We appreciate this suggestion and carefully considered whether the Results section could be shortened. We believe the current level of detail is necessary to ensure findings are accurately interpreted and key methodological information is retained (in particular, the presentation of nonlinear relationships and sensitivity analyses). We have therefore retained the overall structure of this section to support transparency and align with STROBE reporting guidelines.	n/a

Reviewer 2: We note Reviewer 2's comment but in the absence of more explicit guidance on what specifically must be addressed, we trust that our responses to comments from the Editor, Reviewer 1, and Reviewer 3 will address any concerns.

**Reviewer 3**		
Comment	Authors' Response	Page

The abstract is very clear but two details could be added: The study dates, and the effective N (7,515 nurses in 95 wards).	We thank the reviewer for this comment and have added these details to the Abstract (participants heading): "**Registered nurses (N=7,515) working across 95 adult acute wards in 2015-2020.**"	1
"...had the higher odds for sickness (OR 1.68 and 1.07 respectively, p<0.001)" I suggest rephrasing to make this clearer, for example, "were associated with increased odds of sickness"	We have used this suggestion for making this text more concise, thank you.	1
The methods would generally be clearer if STROBE-like headings were used, e.g. study design, setting, participants, variables, so that readers can easily locate information.	Thank you for this suggestion. We agree that clearer subheadings will improve the navigability of the Methods section. We have introduced the following STROBE-informed headings and rearranged Methods content accordingly: **2.1 Study Design, Setting, Participants; 2.2 Variables; 2.3 Statistical Methods, Sensitivity Analysis**	3-4
Repeated measures appear to be handled by including a random intercept of nurse ID, but this is not directly stated in the methods. The description is slightly ambiguous, with separate mentions of a random intercept and random effect. It would be helpful to directly define the specification of the mixed effects model. The ICC justification is somewhat unnecessary, because the data contains non-independent repeated measures which would violate the assumptions of standard regression models, so this data requires a mixed model regardless of ICC.<br><br>The nurses were from 95 different wards. It is possible that there could be variation in both shift patterns and sickness by ward due to differences in practice, which could introduce ward-level clustering. This could be explored descriptively and if there is evidence of clustering, accounting for it with another random intercept or as a fixed effect (covariate adjustment). With so many wards this may not be feasible (e.g. low power, poor model convergence) but it would be useful to explore and discuss - even if it is just to confirm there is no evidence of clustering.	Thank you for these comments. We agree that ward-level variation in sickness absence could plausibly contribute to clustering. Ward-level clustering was assessed as part of our original analysis; we have now made this explicit in the Methods section: **"We used random intercept logistic mixed models to estimate the association between shift pattern variables and sickness absence. Repeated shift-level observations were clustered within nurses and accounted for by using nurse-level random intercepts. Intraclass correlation coefficients (ICC) were calculated to quantify clustering within nurses and wards. Significant clustering was observed at the nurse-level (Adjusted ICC = 0.710) but not at the ward-level; therefore, additional ward-level effects were not included in models."**	4

To help prepare readers and align with STROBE I recommend adding a full specification of the multivariable model: the outcome, exposure, and covariates. There is mention of control variables but listing these and their purpose (to minimize confounding or improve precision?), would ensure this is fully transparent.	Thank you for this comment. We have revised the Methods section to explicitly list exposure variables and covariates included in multivariable analyses, and clarified that covariates were included to minimise potential confounding:<br>"We used random intercept logistic mixed models to estimate the association between shift pattern variables **and the outcome of** sickness absence**."**<br>"Specifically, our **main exposure** variables included...(bulleted list)"<br>"We also included other working time-related **covariates that may confound nurses' sickness absence**...(list)"	3-4
Multivariable models with the smallest Akaike information criterion (AIC) and Bayesian information criterion (BIC) values were selected for interpretation." It sounds as if most elements of the model are fixed based on prior theory. Which aspects were allowed to vary and were optimized based on AIC/BIC?	We recognise that this wording may have implied more specific model comparison/selection than was undertaken. Exposure and control variables were selected a priori based on established frameworks and previous literature (details included on Methods section 2.2, Variables). Section 2.3 has now been revised to clarify that: "Akaike information criterion (AIC) and Bayesian information criterion (BIC) values were **used to compare alternative model specifications, including exposure window lengths and quadratic/cubic term forms, with lower values indicating improved model fit**."	4
The tables could be improved by adding the N, as some may differ from the main N - e.g. how many are full-time/part-time, how many had adequate data for multivariable modelling.	We appreciate the reviewer highlighting the importance of transparency regarding sample size across analyses. Relevant nurse and linked shift counts are currently provided in Section 3.1 (Descriptive Statistics). Additionally, all available records and eligible exposure windows were retained in modelling (following the exclusions described in section 2.2 Variables); we have added the following to make this more explicit: "**Following this predefined exclusion, all records and exposure windows were retained in models**."	4
Table 2: The table instructs readers to refer to figure 1 for the multivariable results of the proportional results, but this figure does not show the uncertainty which makes it difficult to interpret.	Thank you for highlighting this, we agree that providing 95% confidence intervals for each proportion reported will help readers to interpret results. We have calculated and added these values to Table 4, Figures 1 and S1 (as error bars), as well as narratively within the results section.	6, Figure 1, Figure S1
Table 2: Please add specific p-values where they are not too small, as this is recommended in STROBE.	We have updated all relevant tables to include specific p values when $p>0.001$ (otherwise left as $p<0.001$) in the main document and the supplementary material; thank you for flagging this.	5-6, Sup. material

Collinearity is not commented on in the results, but the model in table 2 contains a lot of exposures or covariates that overlap conceptually or possibly even mathematically. Almost all were found to be significant which perhaps indicates this is not a major issue. But if there are exposures which overlap substantially, or even occur downstream from one another, this could split the effect of interest, which would bias the odds ratio towards the null. The results could comment on collinearity results in the methods, and/or add rationale to the methods for modelling all exposures together.	Thank you for this observation. Multicollinearity was assessed using VIF; all nonpolynomial predictors demonstrated low collinearity (VIF < 3 for the majority of variables). Elevated VIFs for polynomial terms were expected, as these terms were derived from the same underlying variable and were therefore not considered indicative of problematic collinearity. We have added clarification to Results section 3.2 to report this explicitly: "In the full multivariable model (Table 2), **multicollinearity was low across all predictors (VIF < 5, excluding polynomial terms where elevated values were expected)**."	5
The interpretation is reasonable but there is occasional causal language (negative effects for example) which go beyond what the data can demonstrate - the data was observational and with limited individual-level data to minimize potential confounding effects. Otherwise the discussion is strong, and my only minor suggestion is to comment on whether or not ward-based clustering was plausible and would impact the interpretation.	We appreciate the reviewer highlighting this; although the term "effects" was intended in a statistical sense, we recognise that some phrasing could be read as causal given our study design. We have carefully reviewed and revised the Discussion section to use more cautious language, for example, referring to associations and higher or lower odds rather than "effects".	7-9

We look forward to hearing from you in due time regarding our revised manuscript and are happy to respond to any further questions or comments that may arise.
