# Supplementary material for: Associations between adverse working hours and nurses’ sickness absence: a longitudinal analysis of e-roster data from acute hospital wards
Source: BMJ Open. 2026 Jul 21;16(7):e120066. doi: 10.1136/bmjopen-2026-120066 (PMC13410695; doi:10.1136/bmjopen-2026-120066)

BMJ Open

BMJ Open is committed to open peer review. As part of this commitment we make the peer review history of every article we publish publicly available.

When an article is published we post the peer reviewers' comments and the authors' responses online. We also post the versions of the paper that were used during peer review. These are the versions that the peer review comments apply to.

The versions of the paper that follow are the versions that were submitted during the peer review process. They are not the versions of record or the final published versions. They should not be cited or distributed as the published version of this manuscript.

BMJ Open is an open access journal and the full, final, typeset and author-corrected version of record of the manuscript is available on our site with no access controls, subscription charges or pay-per-view fees (http://bmjopen.bmj.com).

If you have any questions on BMJ Open's open peer review process please email info.bmjopen@bmj.com

BMJ Open

Associations between adverse working hours and nurses' sickness absence: a longitudinal analysis of e-roster data

Journal:	*BMJ Open*
Manuscript ID	bmjopen-2026-120066
Article Type:	Original research
Date Submitted by the Author:	23-Mar-2026

Complete List of Authors:	Emmanuel, Talia ; University of Southampton, School of Health Sciences<br>Griffiths, Peter; University of Southampton, School of Health Sciences; NIHR Applied Research Collaboration Wessex<br>Lamas-Fernandez, Carlos; University of Southampton, Southampton Business School<br>Dall'Ora, Chiara; University of Southampton, School of Health Sciences; NIHR Applied Research Collaboration Wessex

Keywords:	Nurses, Health Workforce, Organisation of health services < HEALTH SERVICES ADMINISTRATION & MANAGEMENT

SCHOLARONE™ MANUSCRIPTS

BMJ

I, the Submitting Author has the right to grant and does grant on behalf of all authors of the Work (as defined in the below author licence), an exclusive licence and/or a non-exclusive licence for contributions from authors who are: i) UK Crown employees; ii) where BMJ has agreed a CC-BY licence shall apply, and/or iii) in accordance with the terms applicable for US Federal Government officers or employees acting as part of their official duties; on a worldwide, perpetual, irrevocable, royalty-free basis to BMJ Publishing Group Ltd ("BMJ") its licensees and where the relevant Journal is co-owned by BMJ to the co-owners of the Journal, to publish the Work in this journal and any other BMJ products and to exploit all rights, as set out in our licence.

The Submitting Author accepts and understands that any supply made under these terms is made by BMJ to the Submitting Author unless you are acting as an employee on behalf of your employer or a postgraduate student of an affiliated institution which is paying any applicable article publishing charge ("APC") for Open Access articles. Where the Submitting Author wishes to make the Work available on an Open Access basis (and intends to pay the relevant APC), the terms of reuse of such Open Access shall be governed by a Creative Commons licence – details of these licences and which Creative Commons licence will apply to this Work are set out in our licence referred to above.

Other than as permitted in any relevant BMJ Author's Self Archiving Policies, I confirm this Work has not been accepted for publication elsewhere, is not being considered for publication elsewhere and does not duplicate material already published. I confirm all authors consent to publication of this Work and authorise the granting of this licence.

For peer review only

Associations between adverse working hours and nurses' sickness absence: a longitudinal analysis of e-roster data

Talia EMMANUEL[1*], Peter GRIFFITHS[1,2], Carlos LAMAS-FERNANDEZ[2,3], Chiara DALL'ORA[1,2]

[1]School of Health Sciences, University of Southampton, Southampton, UK
[2]National Institute for Health Research (NIHR) Applied Research Collaboration, Wessex, UK
[3]Southampton Business School, University of Southampton, Southampton, UK
*Corresponding author, at T.Emmanuel@soton.ac.uk, https://orcid.org/0000-0001-5595-685X

ABSTRACT:

Objective: To examine the associations between registered nurses' shift work patterns and sickness absence in hospital inpatient wards.

Design: Retrospective longitudinal study using 1.4 million e-roster and sickness absence records analysed with logistic mixed-effects models.

Setting: All inpatient hospital wards in two large health care organisations (Trusts) in England.

Participants: All registered nurses working in adult acute care settings.

Primary Outcome Measure: Nurses' sickness absence in relation to the shift pattern configurations worked in the previous 7 days.

Results: Higher odds for the start of a sickness episode were significantly associated with nurses having rest periods of fewer than 11 hours (OR 1.23, 95% CI 1.19-1.27, p<0.001), more frequent changes between day and night shifts (OR 1.09, 95%CI 1.04-1.13, p<0.001), and spells of 3 or more consecutive shifts (OR 1.24, 95% CI 1.16-1.32, p<0.001) in the previous 7 days. By accounting for nonlinearity, analyses revealed that working >70% of shifts as long shifts and 100% of shifts as night shifts had the higher odds for sickness (OR 1.68 and 1.07 respectively, p<0.001). Sensitivity analysis using 28-day exposure windows did not change direction of effects, however the magnitude and significance of some associations attenuated.

Conclusions: This study highlights the organisational consequences of adverse working hours arrangements for registered nurses working in hospital wards. Short recovery periods, frequent shift rotations, consecutive working spells, and high proportions of long and night shifts were associated with higher odds of sickness absence, with associations most apparent over 7-day exposure windows. Overall, these findings can inform shift planning policies that better support staff wellbeing through proactive management of sickness absence.

STENGTHS AND LIMITATIONS OF THIS STUDY:

- Analysis of registered nurses' shift working patterns from comprehensive dataset of electronic roster records from two large health organisations
- Linkage of roster and sickness data allowed working hours exposures to be examined in direct relation to the onset of sickness absence

For peer review only

For peer review only - http://bmjopen.bmj.com/site/about/guidelines.xhtml

- Mixed-effects modelling that captured non-linear features of recent shift scheduling and repeated observations within individual nurses
- To offset absence of demographic information and inconsistent records of overtime, other variables such as prior sickness absence and total hours worked were included in models

1. INTRODUCTION

In health care settings such as hospitals, nursing staff are routinely required to work non-standard hours (i.e., outside of 07:00 and 19:00, Monday-Friday), extended work shifts (e.g., working for 12 hours or longer), and rotating day-night schedules. These patterns of work are inherently disruptive to personal circadian rhythms, with implications for physiological functions such as hormone release, sleep-wake cycles, and metabolism (1). Consistent with this, there is a wealth of international evidence that links shift work with negative outcomes for staff, including increased fatigue and burnout, poor work-life balance, and development of chronic illness or cancer in the long-term (2-5). Health care employers therefore have a duty to ensure that working hours are organised in ways that minimise these risks and protect the wellbeing of their nursing workforce.

One organisational outcome useful for monitoring workforce wellbeing is staff sickness absence, as documented by historical rosters and/or payroll records. Administrative records of shifts cancelled due to sickness absence offer a readily accessible interpretation of staff wellness: when calling in sick, it can be inferred that most staff do not feel able to work because they are not well. Furthermore, significant upticks in rates of sickness absence among different working environment exposures can provide clues as to where targeted strategies for improvement are warranted. Elevated sickness absence rates place considerable strain on health care organisations (6) and can force reliance on costly temporary staffing or worsen patient wellbeing and other measures when staffing levels fall below planned thresholds (7, 8).

Recent national data on sickness absence rates among registered nurses working in England's National Health Service (NHS) show some concerning trends in this regard (9). These data reveal higher levels of sickness when compared to those prior to the start of the COVID-19 pandemic, as well as when compared to other health professions and the public sector overall. The most common recorded reasons for sickness absence (when measured by percentage of full-time equivalent (FTE) days lost due to sickness) were related to anxiety, stress, depression, and other psychiatric illness (25%) (9). Furthermore, 46% of registered nurses responding to the annual staff survey conducted by the NHS (10) reported feeling unwell as a result of work-related stress specifically over the last 12 months. Although preventing all sickness absence is not possible, any harmful contribution of nurses' working environments should be minimised.

Previous research analysing administrative records of shifts and sickness absence have shown increased rates when nursing staff are working certain configurations of shifts, including long shifts (i.e., ≥12 hours), night shifts, long weeks (i.e., ≥48 working hours per week), and quick returns (i.e., <11 hours of inter-shift recovery time) (11-15). However, there is a gap in understanding the effects of cumulative patterns of work and recovery, particularly those that occur across multiple days. The present study builds on this previous

For peer review only - http://bmjopen.bmj.com/site/about/guidelines.xhtml

research by examining how sequences of shift intensity across consecutive days, total weekly working hours, and inter-shift rest periods are associated with nurses' sickness absence.

2. METHODS

We conducted a retrospective longitudinal analysis of historical shift and sickness absence data recorded in electronic staff rostering systems from all adult acute inpatient wards in two NHS hospital Trusts in England. Original data were collected and anonymised as part of a larger project exploring the staff-, patient-, and cost-related consequences of different health care staffing configurations (16). Administrative pay bands were used to identify registered nurses (band 5 or above). Unique study identifiers were used to link shifts and sickness episodes to the same nurse across the study period, and therefore all variables and analyses were calculated at the shift-per-nurse level. Demographic information for staff (e.g., age, year join/left hospital, number of years in current role) were not available due to data governance restrictions from participating hospitals. Shifts that were not worked due to sickness were aggregated into episodes, starting on the first day that a nurse was absent from work and finishing on the day they returned for at least one shift. Ethical approval was obtained from the NHS Health Research Authority and the University of Southampton Ethics Committee; the requirement for informed consent was waived as all supplied data were de-identified (anonymous).

For the present study, we analysed the roster and sickness absence records of all registered nurses scheduled to work on wards between April 2015 and September 2020. From these data, we created a series of shift pattern variables that accounted for the following configurations: long working hours, night work, spells of consecutive working days, inadequate recovery time, and shift rotations. Each variable was defined by a 7-day exposure period, i.e. the measurements taken in rolling windows of 7 days prior to each worked shift and sickness absence episode. These variables were created in accordance with an established framework for characterising working time exposure in relation to staff wellbeing (17), as well as with nurses' more recent reports of the difficulties of working several consecutive shifts or transitioning from night to day duties with inadequate recovery time (18, 19). Specifically, our main variables included:

- Proportion of shifts worked as long shifts (shifts lasting 12 hours or more)
- Proportion of shifts worked during the night (shifts that finish at 08:00 or earlier)
- Number of spells of consecutive shifts, i.e., 'long' spells (≥ 6 consecutive shifts) and 'intense' spells (≥3 consecutive long or night shifts)
- Number of inadequate rest periods, i.e., 'quick' (≤11.5 hours rest between consecutive shifts) and 'short' returns (≤48 hours rest between a night-to-day shift rotation)
- Number of shift rotations (including night-to-day and day-to-night rotations)

Proportions were used to analyse exposure to long working hours and night work to enable consistent comparison of exposure effects across lookback windows with varied total working hours. Initial exploratory (categorical) analysis of these variables indicated that relationships may not be linear, we therefore added quadratic and cubic terms to our models. We also included other working time-related factors that may influence sickness absence: total number of working hours and total number of hours worked as bank (i.e.,

For peer review only - http://bmjopen.bmj.com/site/about/guidelines.xhtml

when working shifts that cover temporary shortfalls in settings that are different to one's "home" role or ward). We controlled for the number of previous sickness absence episodes, as well as part-time status, or working fewer than a median of 0.75 FTE (full time equivalent) hours per week in the previous quarter (i.e., median of ≤26 hours per week in the previous 13 weeks) (20). Sickness episodes that were preceded by zero working hours in the previous month were removed.

We used random intercept logistic mixed models to estimate the association between shift pattern variables and sickness absence. Given the hierarchical nature of the data, intraclass correlation coefficients (ICC) were calculated to determine the presence of significant clustering behaviour in the outcome; clustering was detected on the nurse-level and was therefore included as random effects in all models. We tested the influence of shift pattern variables on nurses' sickness absence via: univariable models, which examined each main shift pattern variable independently, and full multivariable models, which included all shift pattern and control variables. Multivariable models with the smallest Akaike information criterion (AIC) and Bayesian information criterion (BIC) values were selected for interpretation.

To test for multicollinearity between predictors, we calculated variance inflation factors (VIF), where VIF values <5 indicated low multicollinearity (21). Two sensitivity analyses were conducted: 1) testing variables with 28-day lookback windows to assess whether any observed effects persisted over a longer exposure period, and 2) re-analysing with pre-March 2020 data exclusively to compare and examine if associations were influenced by the onset of the COVID-19 pandemic in England. Shift variables were created with the *pandas* (22) and *datetime* python packages; modelling was undertaken using the *lme4* package in R (version 4.4.0) (23).

3. RESULTS

3.1. Descriptive Statistics

The final dataset contained 1,367,497 worked shifts and 19,876 sickness absence episodes from 7,515 registered nurses across 95 wards. The majority of worked shifts were from nurses working full-time (3789 nurses working 821,681 shifts (60%)) and sickness episodes lasted a median of 4 days long (IQR 2-8 days). The average number/proportion of each shift pattern configuration for full-time and part-time nurses for the entire study period is shown in Table 1. Median shift length and the proportion of long shifts worked were similar between the groups, however full-time nurses worked more night shifts, quick returns, and shift rotations compared to part-time nurses. Part-time nurses had greater variability in their schedules, suggesting more heterogeneous work patterns.

Table 1. Shift patterns worked by full-time (FT) and part-time (PT) nurses in the previous 7 days

Shift Variables	FT Nurses		PT Nurses	
	Mean (SD)	Median	Mean (SD)	Median
**Total hours**	34.3 (5.0)	37.5	23.3 (9.5)	25.0
**N shifts**	2.9 (0.5)	3.0	2.0 (0.8)	2.0
**Average shift length**	12.0 (1.1)	12.5	11.7 (1.4)	12.5
**N long shifts**	2.5 (0.7)	3.0	1.7 (0.9)	2.0

For peer review only - http://bmjopen.bmj.com/site/about/guidelines.xhtml

**Proportion long shifts**	0.9 (0.2)	1.0	0.8 (0.3)	1.0
**N night shifts**	1.0 (0.8)	1.0	0.7 (0.6)	0.0
**Proportion night shifts**	0.3 (0.3)	0.3	0.3 (0.0)	0.0
**N long spells**	0.0 (0.0)	0.0	0.0 (0.0)	0.0
**N intense spells**	0.1 (0.1)	0.0	0.1 (0.1)	0.0
**N quick returns**	0.9 (0.4)	1.0	0.5(0.4)	0.0
**N short returns**	0.2 (0.2)	0.0	0.1(0.1)	0.0
**N shift rotations**	0.7 (0.4)	1.0	0.4 (0.4)	0.0

Supplementary table S1 presents a yearly snapshot of the shift pattern variables worked by full-time nurses specifically. Statistics demonstrate a stable pattern over the 5-year study period. Long (≥6 shifts) and intense (≥3 long or night shifts) consecutive spells were rare across the dataset, however, a slight increase in counts for the latter is noted from 2019 onwards. Similar increases are seen for the mean number of long shifts (2.4 in 2015 versus 2.5 in 2020), the mean proportion of night shifts (0.3 in 2015 versus 0.4 in 2020), and the number of quick returns (mean of 0.9 in 2015 versus 1.0 in 2020), indicating that these shift configurations became more frequent over the study period.

3.2. Multivariable Models

In the full multivariable model (Table 2), reduced odds of sickness absence were observed among nurses who had experienced a sickness episode within the preceding 7 days. In contrast, other covariates either had negligible or nonsignificant associations with sickness absence. For every intense spell of work, quick return, and shift rotation, there was a 24%, 23% and 9% respective increase in the odds of sickness. Working long consecutive spells of work significantly and considerably decreased odds of sickness (OR=0.09, 95% CI 0.01-0.66, p<0.05), however this shift configuration was rare with only 1937 cases across the dataset.

Table 2. Shift pattern configurations worked in the previous 7 days and odds of sickness

**Variables**	**Univariable Models**		**Multivariable Model**	
	**OR (95% CI)**	**Sig**	**OR (95% CI)**	**Sig**
Main Shift Variables				
Proportion long shifts	0.80 (0.75-0.85)	***	(refer to Figure 1)	***
Proportion night shifts	1.06 (1.01-1.11)	*	(refer to Figure 1)	***
N long spells	0.05 (0.01-0.38)	**	0.09 (0.01-0.66)	*
N intense spells	1.01 (0.96-1.06)	n.s.	1.24 (1.16-1.32)	***
N quick returns	0.97 (0.95-0.99)	**	1.23 (1.19-1.27)	***
N short returns	0.94 (0.90-0.98)	**	1.05 (0.99-1.11)	n.s.
N shift rotations	0.94 (0.92-0.96)	***	1.09 (1.04-1.13)	***
Covariates				
Total hours	0.99 (0.99-0.99)	***	0.98 (0.97-0.98)	***
Total bank hours	0.94 (0.94-0.94)	***	0.94(0.94-0.95)	***
N sickness episodes	0.57 (0.51-0.64)	***	0.56 (0.50-0.63)	***
Part-time status	1.07 (1.03-1.11)	***	1.00 (0.96-1.04)	n.s.
			**AIC** 145645	
			**BIC** 145849	

* significant at p<0.05, ** significant at p<0.01, *** significant at p<0.001

For peer review only - http://bmjopen.bmj.com/site/about/guidelines.xhtml

Furthermore, all terms for proportion of long shifts (linear, quadratic, cubic) and proportion of night shifts (linear, quadratic) returned as statistically significant (p<0.001) (Table 3). Table 4 and Figure 1 show how odds of sickness fluctuated across proportions of long shifts and night shifts. Working >30% of shifts as long increased odds for sickness (relative to working no long shifts), with the highest odds observed around the 70% mark (OR=1.68); thereafter, odds progressively declined and fell below 1 at the 100% mark. Working approximately >90% of shifts as night also slightly increased odds for sickness (relative to working no night shifts), with the highest odds occurring at the 100% mark (OR=1.07).

Table 3. Nonlinear terms for proportion of long shifts and night shifts in the previous 7 days

Variable	Term	β	SE	Sig
Proportion long shifts	Linear	-2.09	0.44	***
	Quadratic	8.73	1.12	***
	Cubic	-6.69	0.70	***
Proportion night shifts	Linear	-0.44	0.13	**
	Quadratic	0.50	0.14	***

* significant at p<0.05, ** significant at p<0.01, *** significant at p<0.001

Table 4. Plotting values for nonlinear curves – proportion of long shifts and night shifts in the previous 7 days

	Prop long shifts		*Prop night shifts*	
**Proportion**	**Combined β terms**	**OR**	**Combined β terms**	**OR**
0	0.00	1.00	0.00	1.00
0.1	-0.13	0.88	-0.04	0.96
0.2	-0.12	0.88	-0.07	0.93
0.3	-0.02	0.98	-0.09	0.92
0.4	0.13	1.14	-0.09	0.91
0.5	0.30	1.35	-0.09	0.91
0.6	0.44	1.55	-0.08	0.92
0.7	0.52	1.68	-0.06	0.94
0.8	0.49	1.63	-0.03	0.97
0.9	0.31	1.36	0.01	1.01
1	-0.06	0.95	0.07	1.07

For peer review only - http://bmjopen.bmj.com/site/about/guidelines.xhtml

For peer review only

Figure 1. Cubic and quadratic curves for proportion of long shifts and night shifts in the previous 7 days

[Insert Figure 1 here]

As a sensitivity analysis, we re-estimated associations using a 28-day exposure window, the results of which are reported in Table S2, S3, and Figure S1. The 28-day multivariable model showed higher AIC and BIC values, indicating that the 7-day exposure window model provided a more parsimonious fit. For most shift pattern variables, the direction of effects was the same across the two exposure windows (i.e., increased odds for sickness absence), although effect sizes were attenuated in the 28-day model. Both measures of consecutive working spells were not statistically significant in the 28-day model, indicating that the influence of this element of shift intensity was more pronounced over weekly timeframes. Further sensitivity analyses restricting data to pre-March 2020 records showed minimal differences in effect sizes (i.e., consistent direction and magnitude of effects with similar confidence intervals), confirming that our primary findings were not influenced by any unrealised, pandemic-related changes in working hours configurations.

4. DISCUSSION

The purpose of this observational study was to gain a comprehensive understanding of how adverse working time configurations contribute to sickness absence rates among registered nurses. This was achieved by analysing 1.4 million historical shift and sickness absence records collected from acute inpatient wards in two NHS hospital Trusts between 2015 and 2020. Compared to previous research on the impact of shift types on indicators of workforce wellbeing, this analysis explored the cumulative influence of patterns of work and rest, showing that higher proportions of long hours and night work, quick returns (having fewer than 11 hours of inter-shift recovery), intense spells of consecutive shifts (≥3 long or night shifts), and frequent shift rotations were associated with increased odds for sickness absence.

In our study, nurses working high proportions of long shifts and night shifts had increased risk of sickness, with the highest odds observed when over 70% of shifts were long (≥12 hours) or when working all night shifts in lookback windows. These findings mirror those of previous studies conducted within England. For example, in an analysis of 601,282 shift records from a large acute hospital, when 75% or more of shifts were worked as long shifts or night shifts in the past 7 days, the odds of sickness absence were increased when compared to working no long shifts (24% increase in odds) or day shifts only (12 % increase in odds) (11, 12). Similarly, in a study that examined a pre-versus-post change in sickness absence rates in a large mental health hospital, an increase in the percentage of sickness hours per week (ranging from 0.73%-0.98%, or,1 shift per ward per week) was found following the organisational implementation of long shifts (15).

However, the nonlinear effects observed in this study provide new and nuanced understanding of these relationships. Although high proportions of long shifts led to increased odds for sickness (relative to working no long shifts), the magnitude of these odds quickly diminished when proportions were greater than 70% and ultimately led to a decrease in odds at the 100% mark. This first appears as counterintuitive given the demanding and fatiguing nature of extended shifts, as evidenced by a substantial body of literature. One

For peer review only - http://bmjopen.bmj.com/site/about/guidelines.xhtml

possible explanation for this observation is that nurses who work most or all long shifts benefit from an element of consistency (i.e., always working the same shift length), and therefore are able to establish routines and coping mechanisms accordingly. Rota consistency has been previously identified by nurses as a key scheduling practice that is supportive of their personal priorities in and outside of work (18), and the benefits of having some consistency may lessen the impact of long working hours. Self-selection similar to the ‘healthy worker effect’ (24) may also help explain this result, as individuals who can work demanding shifts and schedules continue to do so, while those more susceptible to sickness change their working patterns (or switch to standard schedules completely), thus skewing data and relationships toward the appearance of healthier outcomes.

Significant nonlinear effects were also observed for the proportion of night shifts worked in the previous 7 days, where a slight increase in the odds for sickness only resulted from working 100% night shifts (relative to working no night shifts) - a feature of permanent night schedules. While existing guidance around night work advises against permanent night work, an oft-assumed benefit of this shift pattern is the avoidance of routine disruption that arises from rotating shiftwork. Our results contribute to the body of research that challenges this belief, as any benefits derived from consistency in this sense is likely overshadowed by the harmful effects of nightwork in general (e.g., inability to reconcile work routines with personal/social routines, sustained metabolic dysregulation), thus leading to higher rates of sickness. The reduced odds of sickness observed for proportions below 100% may be due to a reluctance to call in sick when scheduled to work night shifts (as part of a mixed day/night pattern) that arises from concerns about leaving wards understaffed or forfeiting pay premiums associated with working unsocial hours (25).

Another high demand shift configuration, intense spells of consecutive work (i.e., working more than 3 long shifts or night shifts consecutively) increased odds for sickness. This finding complements previous research on the consequences of working many consecutive shifts: increased sickness absence rates when health care workers are working ≥ 4 or 5 consecutive night shifts, as well as consequences to other outcomes such as cognition, performance, occupational injury rates, and sleepiness when nurses are working consecutive long shifts (26-28). However, results from qualitative research are mixed, with some nurses voicing their preference for consecutive shifts to enable longer periods of uninterrupted time off, while others call out the challenges of excessive consecutive shifts that impede one’s ability to engage in life outside of work as a result of exhaustion or fatigue (18, 29).

The negative effects of working consecutive shifts did not persist when examining long spells of continuous work (i.e., working more than 6 consecutive long or night shifts) as this variable significantly and considerably decreased odds for sickness in multivariable models. This configuration was rare across the dataset (<0.1%), which may indicate that when a long spell was worked, it was done so out of choice. Previous research suggests that choice and autonomy may influence the relationship between the demands of shift work and staff wellness outcomes (30, 31), particularly for sickness absence as nurses may be less likely to call in sick for shift patterns they chose or requested. Other variables tested in our model that reflected some element of agency, such as the total number of bank hours and the number of sickness episodes in the lookback window, also significantly decreased odds of sickness. For this latter variable however, “agency” does not necessarily reflect making active choices over working time but rather could represent behavioural responses to

For peer review only - http://bmjopen.bmj.com/site/about/guidelines.xhtml

absence monitoring policies enforced by employers. For example, some NHS Trusts make use of the controversial Bradford scoring tool (32), which penalises recurrent and short absences and thereby discourages employees from taking frequent sick leave, even when clinically necessary.

Lastly, shift configurations involving inadequate rest periods, namely quick returns and shift rotations, also significantly increased the odds of sickness. Of note, these findings were observed even though both variables were defined broadly to capture all relevant cases in the study population (i.e., including both day-to-night and night-to-day changes, defining quick returns as fewer than 11.5 hours of rest between shifts to account for the pervasive use of long shifts). Similar findings have been found in previous register-based sickness absence studies (13, 33), as well as in other research exploring outcomes such as stress, exhaustion and fatigue (34). Nevertheless, short returns – which represent a special case of inadequate rest (<48 hours) between a night-to-day shift rotation – did not return significant results in our model. Having limited rest time between ending a night shift and starting a day shift has previously been identified as a problematic work/rest day configuration by nurses (18); therefore, while this shift pattern may negatively impact overall wellbeing and work-life balance in some ways, it does not seem to directly influence sickness as recorded through official mechanisms.

4.1. Limitations

The absence of demographic information in the underlying dataset (e.g., age, years in current role, active flexible working requests) restricted the ability to control for variables that may also impact odds of sickness absence. However, these confounding effects were at least partially controlled for by accounting for outcome clustering at the individual/nurse level and other variables such as number of hours worked as 'bank' and the number of previous sickness episodes. Second, overtime work (working in excess of 37.5 hours per calendar week when averaged over a reference period) (NHS Employers, 2024) is another shift variable previously shown to impact staff wellbeing, particularly when worked in excess of a working week with long and/or night shifts (Health and Safety Executive (HSE), 2006). However, this variable could not be reliably assessed in the data, as elements of overtime work (e.g., extra hours worked at the end of a planned shift versus additional shifts that are worked in excess of the full-time limit) were recorded inconsistently. This limitation was in part mitigated by the inclusion of total working hours as a controlling variable, which also effectively captured extra work time that may not have been formally recorded as overtime.

5. CONCLUSIONS

This study of historical shift records provides detailed insight into the link between working hours and sickness absence among registered nurses working in acute adult hospital wards. Over the 5-year study period, changes in shift patterns were detected, with notable increases in configurations such as spells of consecutive working days and inadequate inter-shift recovery. These configurations were also associated with higher odds of sickness, with the number of intense spells, quick returns, and shift rotations demonstrating effects in 7-day exposure windows. High proportions of long shifts and night shifts also significantly increased sickness absence, though these relationships were nonlinear and may have been influenced by other factors such as personal adaptability to adverse working conditions and

For peer review only - http://bmjopen.bmj.com/site/about/guidelines.xhtml

discretion used when taking sick leave. Our findings highlight the organisational consequences of adverse working hours arrangements and can inform shift planning strategies that prioritise nurse wellbeing and proactive management of sickness absence.

For peer review only

For peer review only - http://bmjopen.bmj.com/site/about/guidelines.xhtml

FUNDING: The study dataset was derived from a project funded by the UK National Institute for Health and Care Research (NIHR) Health Services and Delivery Research Programme (award No. NIHR128056) and the NIHR Applied Research Collaboration (Wessex). The primary author was supported by the UKRI Economic and Social Research Council South Coast Doctoral Training Partnership (Grant Number ES/P000673/1). The views expressed are those of the author(s) and not necessarily those of the NIHR or the Department of Health and Social Care. These funders had no role in the design, conduct, or reporting of the study.

DECLARATION OF INTEREST: No competing interests to declare.

DATA AVAILABILITY: Due to the data sharing agreements with the providers, we are unable to share the source data.

REPORTING: This study is reported in accordance with the Strengthening the Reporting of Observational Studies in Epidemiology (STROBE) guidelines.

AUTHOR CONTRIBUTIONS: Talia Emmanuel: Conceptualisation, Formal analysis, Writing (original draft). **Peter Griffiths**: Conceptualisation, Methodology, Project administration, Formal analysis, Writing (original draft). **Carlos Lamas-Fernandez**: Formal analysis, Writing (original draft). **Chiara Dall'Ora**: Conceptualisation, Methodology, Project administration, Formal analysis, Writing (original draft).

For peer review only

REFERENCES

1. Health and Safety Executive (HSE). Managing shiftwork - Health and safety guidance. 2006.
2. Grzywacz JG. Shift Work and Its Implications for Everyday Work and Family Life: A Foundation and Summary. In: Iskra-Golec I, Barnes-Farrell J, Bohle P, editors. Social and Family Issues in Shift Work and Non Standard Working Hours. Cham: Springer International Publishing; 2016. p. 3–17.
3. Arlinghaus A, Bohle P, Iskra-Golec I, Jansen N, Jay S, Rotenberg L. Working Time Society consensus statements: Evidence-based effects of shift work and non-standard working hours on workers, family and community. Ind Health. 2019;57(2):184–200.
4. Moreno CRC, Marqueze EC, Sargent C, Wright Jr KP, Ferguson SA, Tucker P. Working Time Society consensus statements: Evidence-based effects of shift work on physical and mental health. Industrial Health. 2019;57(2):139–57.
5. Dall'Ora C, Ball J, Recio-Saucedo A, Griffiths P. Characteristics of shift work and their impact on employee performance and wellbeing: A literature review. International Journal of Nursing Studies. 2016;57:12–27.
6. Johnson CJ, Croghan E, Crawford J. The problem and management of sickness absence in the NHS: considerations for nurse managers. Journal of nursing management. 2003;11(5):336–42.
7. Dall'Ora C, Saville C, Rubbo B, Turner L, Jones J, Griffiths P. Nurse staffing levels and patient outcomes: A systematic review of longitudinal studies. International Journal of Nursing Studies. 2022;134:N.PAG–N.PAG.
8. Senek M, Robertson S, Ryan T, King R, Wood E, Tod A. The association between care left undone and temporary nursing staff ratios in acute settings: a cross-sectional survey of registered nurses. BMC Health Services Research. 2020;20(1):637.
9. NHS Digital. NHS Sickness Absence Rates 2024 [Available from: https://digital.nhs.uk/data-and-information/publications/statistical/nhs-sickness-absence-rates/april-2024.
10. NHS. NHS Staff Survey - National Results Briefing 2026 [updated March 2026. Available from: https://www.nhsstaffsurveys.com/results/national-results/.
11. Dall'Ora C, Ball J, Redfern O, Recio-Saucedo A, Maruotti A, Meredith P, et al. Are long nursing shifts on hospital wards associated with sickness absence? A longitudinal retrospective observational study. Journal of nursing management. 2019;27(1):19–26.
12. Dall'Ora C, Ball J, Redfern OC, Griffiths P. Night work for hospital nurses and sickness absence: a retrospective study using electronic rostering systems. Chronobiology international. 2020;37(9-10):1357–64.
13. Larsen AD, Ropponen A, Hansen J, Hansen ÅM, Kolstad HA, Koskinen A, et al. Working time characteristics and long-term sickness absence among Danish and Finnish nurses: A register-based study. International journal of nursing studies. 2020;112:103639.
14. Ropponen A, Koskinen A, Puttonen S, Härmä M. Exposure to working-hour characteristics and short sickness absence in hospital workers: A case-crossover study using objective data. International Journal of Nursing Studies. 2019;91:14–21.
15. Rodriguez Santana I, Anaya Montes M, Chalkley M, Jacobs R, Kowalski T, Suter J. The impact of extending nurse working hours on staff sickness absence: Evidence from a large mental health hospital in England. International journal of nursing studies. 2020;112:103611.
16. Griffiths P, Dall'Ora C, Saville C, Culliford D, Jones J, Meredith P, et al. Consequences, costs and cost-effectiveness of different workforce configurations in English acute hospitals: a longitudinal retrospective study using routinely collected data: National Institute for Health and Care Research; 2023 [Available from: https://fundingawards.nihr.ac.uk/award/NIHR128056.

For peer review only - http://bmjopen.bmj.com/site/about/guidelines.xhtml

17. Härmä M, Ropponen A, Hakola T, Koskinen A, Vanttola P, Puttonen S, et al. Developing register-based measures for assessment of working time patterns for epidemiologic Studies. Scandinavian Journal of Work, Environment and Health. 2015;41(3):268–79.
18. Emmanuel T, Griffiths P, Lamas-Fernandez C, Ejebu OZ, Dall'Ora C. The important factors nurses consider when choosing shift patterns: A cross-sectional study. Journal of Clinical Nursing. 2024;33(3):998–1011.
19. Barker HR, Griffiths P, Dall'Ora C. "I don't think there's necessarily a one size fits all" negotiating competing priorities in nurse shift scheduling: a qualitative study. BMC nursing. 2025;24(1):1048.
20. Van Bastelaer A, Lemaître G, Marianna P. The definition of part-time work for the purpose of international comparisons. OECD Labour Market and Social Policy Occasional Papers. 1997(22).
21. James G, Witten D, Hastie T, Tibshirani R. An Introduction to Statistical Learning: with Applications in R: Springer New York; 2013.
22. McKinney W. Python for Data Analysis: Data Wrangling with pandas, NumPy, and Jupyter: O'Reilly Media; 2022.
23. Bates D, Mächler M, Bolker B, Walker S. Fitting Linear Mixed-Effects Models Using lme4. Journal of Statistical Software. 2015;67(1):1 – 48.
24. Ritonja J, Aronson KJ, Matthews RW, Boivin DB, Kantermann T. Working Time Society consensus statements: Individual differences in shift work tolerance and recommendations for research and practice. Industrial health. 2019;57(2):201–12.
25. NHS Employers. Unsocial hours payments. 2022.
26. Hopcia K, Dennerlein JT, Hashimoto D, Orechia T, Sorensen G. Occupational injuries for consecutive and cumulative shifts among hospital registered nurses and patient care associates: a case-control study. Workplace health & safety. 2012;60(10):437–44.
27. Hirsch Allen AJ, Park JE, Adhami N, Sirounis D, Tholin H, Dodek P, et al. Impact of work schedules on sleep duration of critical care nurses. American journal of critical care : an official publication, American Association of Critical-Care Nurses. 2014;23(4):290–5.
28. James L, Elkins-Brown N, Wilson M, James SM, Dotson E, Edwards CD, et al. The effects of three consecutive 12-hour shifts on cognition, sleepiness, and domains of nursing performance in day and night shift nurses: A quasi-experimental study. International journal of nursing studies. 2021;123:104041.
29. Ejebu OZ, Dall'Ora C, Griffiths P. Nurses' experiences and preferences around shift patterns: A scoping review. PLoS ONE. 2021;16(8 August).
30. Demerouti E, Bakker AB, Nachreiner F, Schaufeli WB. The job demands-resources model of burnout. Journal of Applied psychology. 2001;86(3):499.
31. Dall'Ora C, Ejebu OZ, Ball J, Griffiths P. Shift work characteristics and burnout among nurses: cross-sectional survey. Occupational Medicine. 2023;73(4):199–204.
32. Taylor S. People Resourcing: Managing Absence. 3rd ed: Chartered Institute of Personnel And Development; 2005.
33. Rosenström T, Härmä M, Kivimäki M, Ervasti J, Virtanen M, Hakola T, et al. Patterns of working hour characteristics and risk of sickness absence among shift-working hospital employees: A data-mining cohort study. Scandinavian Journal of Work, Environment and Health. 2021;47(5):395–403.
34. Min A, Min H, Hong HC. Work schedule characteristics and fatigue among rotating shift nurses in hospital setting: An integrative review. Journal of nursing management. 2019;27(5):884–95.

For peer review only - http://bmjopen.bmj.com/site/about/guidelines.xhtml

Figure 1. Cubic and quadratic curves for proportion of long shifts and night shifts in the previous 7 days

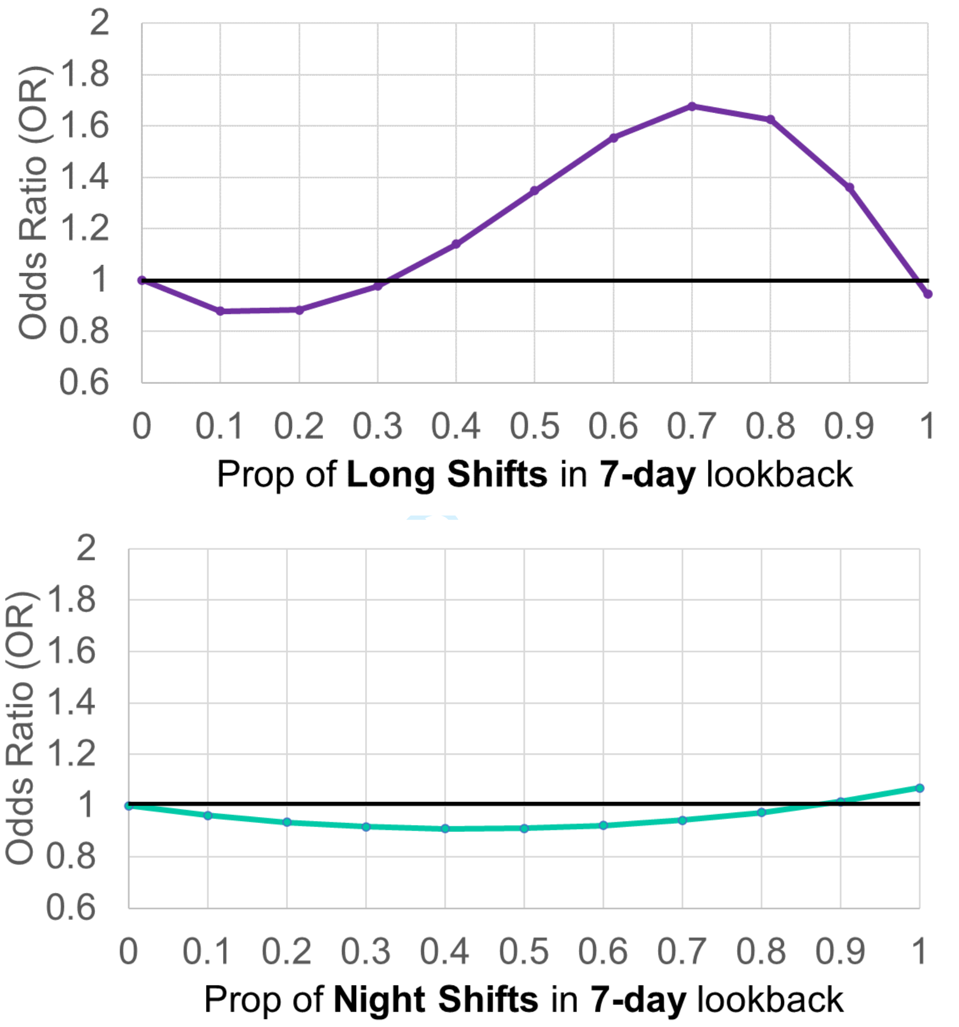

For peer review only - http://bmjopen.bmj.com/site/about/guidelines.xhtml

SUPPLEMENTARY MATERIAL

Supplementary Table S1. Shift pattern configurations worked by full-time nurses in the previous 7 days

Shift Variables	Overall		2015		2016		2017		2018		2019		2020	
	Mean (SD)	Median	Mean (SD)	Median	Mean (SD)	Median	Mean (SD)	Median	Mean (SD)	Median	Mean (SD)	Median	Mean (SD)	Median
Total hours	34.3 (5.0)	37.5	33.9 (4.8)	37.5	33.9 (4.7)	37.5	34.1 (4.5)	37.5	33.9 (4.9)	37.5	34.6 (5.3)	37.5	35.0 (5.6)	37.5
N shifts	2.9 (0.5)	3.0	2.9 (0.5)	3.0	2.9 (0.5)	3.0	2.9 (0.4)	3.0	2.9 (0.5)	3.0	2.9 (0.5)	3.0	2.9 (0.5)	3.0
Avg shift length	12.0 (1.1)	12.5	12.0 (1.1)	12.5	12.0 (1.1)	12.5	12.0 (1.1)	12.5	12.0 (1.1)	12.5	12.0 (1.0)	12.5	12.1 (1.0)	12.5
N long shifts	2.5 (0.7)	3.0	2.4 (0.8)	3.0	2.4 (0.7)	3.0	2.5 (0.7)	3.0	2.4 (0.7)	3.0	2.5 (0.8)	3.0	2.5 (0.8)	3.0
Prop long shifts	0.9 (0.2)	1.0	0.9 (0.3)	1.0	0.9 (0.2)	1.0	0.9 (0.2)	1.0	0.9 (0.2)	1.0	0.9 (0.2)	1.0	0.9 (0.2)	1.0
N night shifts	1.0 (0.8)	1.0	1.0 (0.7)	1.0	1.0 (0.7)	1.0	1.0 (0.7)	1.0	1.0 (0.8)	1.0	1.0 (0.8)	1.0	1.1 (0.8)	1.0
Prop night shifts	0.3 (0.3)	0.3	0.3 (0.2)	0.3	0.3 (0.2)	0.3	0.3 (0.2)	0.3	0.3 (0.3)	0.3	0.4 (0.3)	0.3	0.4 (0.3)	0.3
N long spells	0.0 (0.0)	0.0	0.0 (0.0)	0.0	0.0 (0.0)	0.0	0.0 (0.0)	0.0	0.0 (0.0)	0.0	0.0 (0.0)	0.0	0.0 (0.0)	0.0
N intense spells	0.1 (0.1)	0.0	0.1 (0.1)	0.0	0.1 (0.1)	0.0	0.1 (0.1)	0.0	0.1 (0.1)	0.0	0.2 (0.2)	0.0	0.2 (0.2)	0.0
N quick returns	0.9 (0.4)	1.0	0.9 (0.4)	1.0	0.9 (0.4)	1.0	0.9 (0.4)	1.0	0.9 (0.4)	1.0	1.0 (0.4)	1.0	1.0 (0.5)	1.0
N short returns	0.2 (0.2)	0.0	0.2 (0.2)	0.0	0.2 (0.2)	0.0	0.2 (0.2)	0.0	0.2 (0.2)	0.0	0.2 (0.2)	0.0	0.2 (0.2)	0.0
N shift rotations	0.7 (0.4)	1.0	0.7 (0.4)	1.0	0.7 (0.4)	1.0	0.7 (0.4)	1.0	0.7 (0.4)	1.0	0.7 (0.4)	1.0	0.7 (0.5)	1.0

* significant at p<0.05, ** significant at p<0.01, *** significant at p<0.001

For peer review only

Supplementary Table S2. Shift pattern configurations worked in the previous 28 days and odds of sickness

Variables	Univariable Models		Multivariable Model	
	OR (95% CI)	Sig	OR (95% CI)	Sig
Main Shift Variables				
Prop long shifts	1.11 (1.02-1.20)	*	(refer to Figure S1)	***
Prop night shifts	0.98 (0.91-1.05)	n.s.	(refer to Figure S1)	***
N long spells	0.69 (0.59-0.81)	***	0.98 (0.83-1.16)	n.s.
N intense spells	0.95 (0.93-0.97)	***	0.99 (0.96-1.02)	n.s.
N quick returns	0.98 (0.97-0.99)	***	1.04 (1.02-1.05)	***
N short returns	0.96 (0.94-0.98)	***	0.97 (0.94-1.00)	n.s.
N shift rotations	1.00 (0.99-1.01)	n.s.	1.04 (1.02-1.06)	***
Covariates				
Mean weekly hours	0.99 (0.99-0.99)	***	0.99 (0.99-0.99)	***
Total bank hours	0.98 (0.98-0.98)	***	0.98 (0.98-0.98)	***
N sickness episodes	0.68 (0.65-0.71)	***	0.64 (0.61-0.68)	***
Part-time status	1.07 (1.03-1.11)	***	0.99 (0.95-1.03)	n.s.

* significant at p<0.05, ** significant at p<0.01, *** significant at p<0.001

Supplementary Table S3. Nonlinear terms for proportion of long shifts and night shifts in the previous 28 days

Variable	Term	Beta	Sig
Prop long shifts	Linear	-1.636	***
	Quadratic	6.5484	***
	Cubic	-4.748	***
Prop night shifts	Linear	-0.484	***
	Quadratic	0.546	**

* significant at p<0.05, ** significant at p<0.01, *** significant at p<0.001

For peer review only

Supplementary Figure S1. Cubic and quadratic curves for proportion of long shifts and night shifts in the previous 28 days

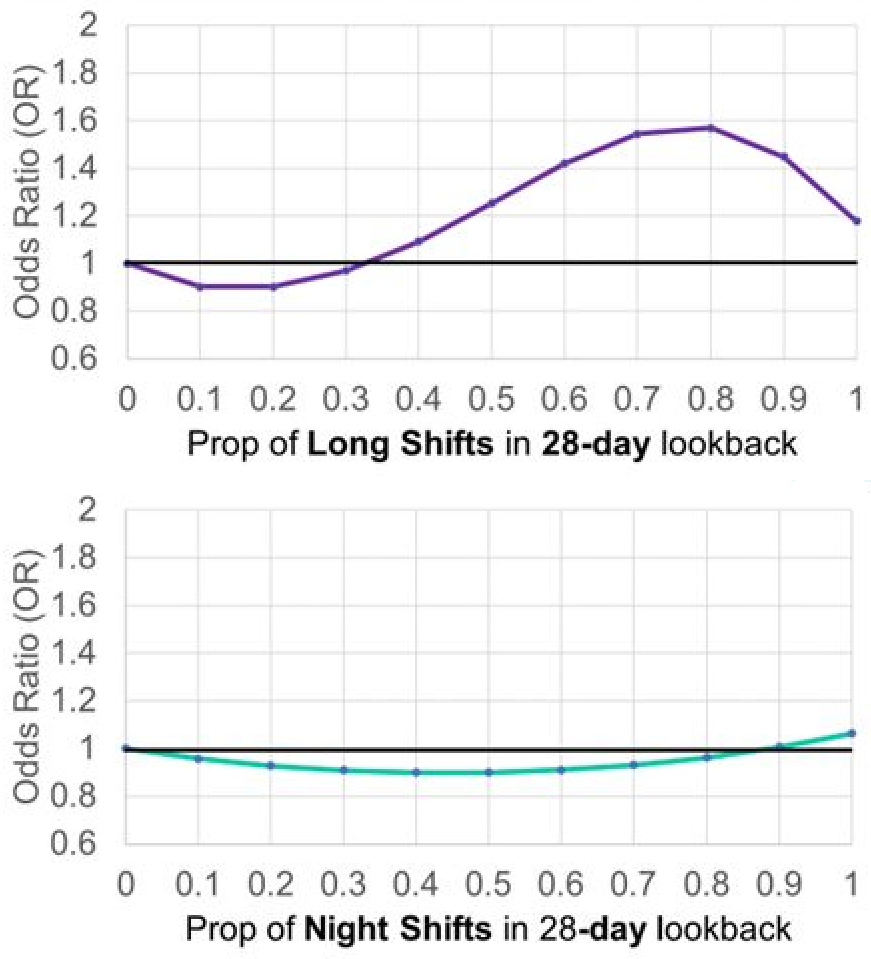

BMJ Open

Associations between adverse working hours and nurses' sickness absence: a longitudinal analysis of e-roster data from acute hospital wards

Journal:	*BMJ Open*
Manuscript ID	bmjopen-2026-120066.R1
Article Type:	Original research
Date Submitted by the Author:	05-Jun-2026

Complete List of Authors:	Emmanuel, Talia ; University of Southampton, School of Health Sciences<br>Griffiths, Peter; University of Southampton, School of Health Sciences; NIHR Applied Research Collaboration Wessex<br>Lamas-Fernandez, Carlos; University of Southampton, Southampton Business School<br>Dall'Ora, Chiara; University of Southampton, School of Health Sciences; NIHR Applied Research Collaboration Wessex

**Primary Subject Heading**:	Health services research
Secondary Subject Heading:	Health services research
Keywords:	Nurses, Health Workforce, Organisation of health services < HEALTH SERVICES ADMINISTRATION & MANAGEMENT

SCHOLARONE™
MANUSCRIPTS

I, the Submitting Author has the right to grant and does grant on behalf of all authors of the Work (as defined in the below author licence), an exclusive licence and/or a non-exclusive licence for contributions from authors who are: i) UK Crown employees; ii) where BMJ has agreed a CC-BY licence shall apply, and/or iii) in accordance with the terms applicable for US Federal Government officers or employees acting as part of their official duties; on a worldwide, perpetual, irrevocable, royalty-free basis to BMJ Publishing Group Ltd ("BMJ") its licensees and where the relevant Journal is co-owned by BMJ to the co-owners of the Journal, to publish the Work in this journal and any other BMJ products and to exploit all rights, as set out in our licence.

The Submitting Author accepts and understands that any supply made under these terms is made by BMJ to the Submitting Author unless you are acting as an employee on behalf of your employer or a postgraduate student of an affiliated institution which is paying any applicable article publishing charge ("APC") for Open Access articles. Where the Submitting Author wishes to make the Work available on an Open Access basis (and intends to pay the relevant APC), the terms of reuse of such Open Access shall be governed by a Creative Commons licence – details of these licences and which Creative Commons licence will apply to this Work are set out in our licence referred to above.

Other than as permitted in any relevant BMJ Author's Self Archiving Policies, I confirm this Work has not been accepted for publication elsewhere, is not being considered for publication elsewhere and does not duplicate material already published. I confirm all authors consent to publication of this Work and authorise the granting of this licence.

For peer review only

Associations between adverse working hours and nurses' sickness absence: a longitudinal analysis of e-roster data from acute hospital wards

Talia EMMANUEL[1*], Peter GRIFFITHS[1,2], Carlos LAMAS-FERNANDEZ[3], Chiara DALL'ORA[1,2]

[1]School of Health Sciences, University of Southampton, Southampton, UK
[2]National Institute for Health Research (NIHR) Applied Research Collaboration, Wessex, UK
[3]Southampton Business School, University of Southampton, Southampton, UK
*Corresponding author, at T.Emmanuel@soton.ac.uk, https://orcid.org/0000-0001-5595-685X

ABSTRACT:

Objective: To examine the associations between registered nurses' shift work patterns and sickness absence in hospital inpatient wards.

Design: Retrospective longitudinal study using 1.4 million e-roster and sickness absence records analysed with logistic mixed-effects models.

Setting: All inpatient hospital wards in two large health care organisations (Trusts) in England.

Participants: Registered nurses (N=7,515) working across 95 adult acute wards in 2015-2020.

Primary Outcome Measure: Nurses' sickness absence in relation to the shift pattern configurations worked in the previous 7 days. Logistic mixed-effects models were adjusted for total working hours, bank hours, previous sickness, and part-time status.

Results: Higher odds of sickness absence were significantly associated with each additional rest period of fewer than 11 hours (OR 1.23, 95% CI 1.19-1.27, $p<0.001$), change between day and night shifts (OR 1.09, 95%CI 1.04-1.13, $p<0.001$), and spell of 3 or more consecutive shifts (OR 1.24, 95% CI 1.16-1.32, $p<0.001$) in the previous 7 days. By accounting for nonlinearity, analyses revealed that working more than 70% of shifts as ≥12-hour shifts and 100% of shifts as night shifts were associated with increased odds of sickness (OR 1.68, 95% CI 1.53-1.84 and OR 1.07, 95% CI 1.01-1.13 respectively) when compared with working none of these shift types. Sensitivity analysis using 28-day exposure windows did not change direction of effects, however the magnitude and significance of some associations attenuated.

Conclusions: This study highlights the organisational consequences of adverse working hours arrangements for registered nurses working in hospital wards. Short recovery periods, frequent shift rotations, consecutive working spells, and high proportions of long and night shifts were associated with higher odds of sickness absence, with associations most apparent over 7-day exposure windows. Overall, these findings can inform shift planning policies that better support staff wellbeing through proactive management of sickness absence.

STRENGTHS AND LIMITATIONS OF THIS STUDY:

- Analysis of registered nurses' shift working patterns from comprehensive dataset of electronic roster records from two large health organisations

For peer review only - http://bmjopen.bmj.com/site/about/guidelines.xhtml

- Linkage of roster and sickness data allowed working hours exposures to be examined in direct relation to the onset of sickness absence
- Mixed-effects modelling that captured non-linear features of recent shift scheduling and repeated observations within individual nurses
- To offset absence of demographic information and inconsistent records of overtime, other variables such as nurses' prior sickness absence and total hours worked in exposure windows were included in models

1. INTRODUCTION

In health care settings such as hospitals, nursing staff are routinely required to work non-standard hours (i.e., outside of 07:00 and 19:00, Monday-Friday), extended work shifts (e.g., working for 12 hours or longer), and rotating day-night schedules. These patterns of work are inherently disruptive to personal circadian rhythms, with implications for physiological functions such as hormone release, sleep-wake cycles, and metabolism [1]. Consistent with this, there is a wealth of international evidence that links shift work with negative outcomes for staff, including increased fatigue and burnout, poor work-life balance, and development of chronic illness or cancer in the long-term [2-5]. Health care employers therefore have a duty to ensure that working hours are organised in ways that minimise these risks and protect the wellbeing of their nursing workforce.

One organisational outcome useful for monitoring workforce wellbeing is staff sickness absence, as documented by historical rosters and/or payroll records. Administrative records of shifts cancelled due to sickness absence offer a readily accessible interpretation of staff wellness: when calling in sick, it can be inferred that most staff do not feel able to work because they are not well. Furthermore, significant upticks in rates of sickness absence among different working environment exposures can provide clues as to where targeted strategies for improvement are warranted. Elevated sickness absence rates place considerable strain on health care organisations [6] and can force reliance on costly temporary staffing or worsen patient wellbeing and other measures when staffing levels fall below planned thresholds [7, 8].

Recent national data on sickness absence rates among registered nurses working in England's National Health Service (NHS) show some concerning trends in this regard [9]. These data reveal higher levels of sickness when compared to those prior to the start of the COVID-19 pandemic, as well as when compared to other health professions and the public sector overall. The most common recorded reasons for sickness absence (when measured by percentage of full-time equivalent (FTE) days lost due to sickness) were related to anxiety, stress, depression, and other psychiatric illness (25%) [9]. Furthermore, 46% of registered nurses responding to the annual staff survey conducted by the NHS (10) reported feeling unwell as a result of work-related stress specifically over the last 12 months. Although preventing all sickness absence is not possible, any harmful contribution of nurses' working environments should be minimised.

Previous research analysing administrative records of shifts and sickness absence have shown increased rates when nursing staff are working certain configurations of shifts, including long shifts (i.e., ≥12 hours), night shifts, long weeks (i.e., ≥48 working hours per week), and quick returns (i.e., <11 hours of inter-shift recovery time) [11-15]. However, there

For peer review only - http://bmjopen.bmj.com/site/about/guidelines.xhtml

is a gap in understanding the effects of cumulative patterns of work and recovery, particularly those that occur across multiple days. The present study builds on this previous research by examining how sequences of shift intensity across consecutive days, total weekly working hours, and inter-shift rest periods are associated with nurses' sickness absence.

2. METHODS

2.1. Study Design, Setting, Participants

We conducted a retrospective longitudinal analysis of historical shift and sickness absence data recorded in electronic staff rostering systems from all adult acute inpatient wards in two NHS hospital Trusts in England. Original data were collected and anonymised as part of a larger project exploring the staff-, patient-, and cost-related consequences of different health care staffing configurations [16]. Administrative pay bands were used to identify registered nurses (band 5 or above). Unique study identifiers were used to link shifts and sickness episodes to the same nurse across the study period, and therefore all variables and analyses were calculated at the shift-per-nurse level. Shifts that were not worked due to sickness were aggregated into episodes, starting on the first day that a nurse was absent from work and finishing on the day they returned for at least one shift. Demographic information for staff (e.g., age, year join/left hospital, number of years in current role) were not available due to data governance restrictions from participating hospitals.

Ethical approval was obtained from the NHS Health Research Authority (ref: 273185) and the University of Southampton Ethics Committee (ref: 52957); the requirement for informed consent was waived as all supplied data were de-identified (anonymous). For the present study, we analysed the roster and sickness absence records of all registered nurses scheduled to work on wards between April 2015 and September 2020.

2.2 Variables

We created a series of shift pattern variables that accounted for the following configurations: long working hours, night work, spells of consecutive working days, inadequate recovery time, and shift rotations. Each variable was defined by a 7-day exposure period, i.e. the measurements taken in rolling windows of 7 days prior to each worked shift and sickness absence episode. This exposure length reflects the weekly structure through which legal and contractual working-time limits are typically defined. Variables were also created in accordance with an established framework for characterising working time exposure in relation to staff wellbeing [17], as well as with nurses' more recent reports of the difficulties of working several consecutive shifts or transitioning from night to day duties with inadequate recovery time [18, 19]. Specifically, our main exposure variables included:

- Proportion of shifts worked as long shifts (shifts lasting 12 hours or more)
- Proportion of shifts worked during the night (shifts that finish at 08:00 or earlier)
- Number of spells of consecutive shifts, i.e., 'long' spells (≥ 6 consecutive shifts) and 'intense' spells (≥3 consecutive long or night shifts)
- Number of inadequate rest periods, i.e., 'quick' (≤11.5 hours rest between consecutive shifts) and 'short' returns (≤48 hours rest between a night-to-day shift rotation)
- Number of shift rotations (including night-to-day and day-to-night rotations)

For peer review only - http://bmjopen.bmj.com/site/about/guidelines.xhtml

Proportions were used to analyse exposure to long working hours and night work to enable consistent comparison of exposure effects across lookback windows with varied total working hours. Initial exploratory (categorical) analysis of these variables indicated that relationships may not be linear, we therefore added quadratic and cubic terms to our models.

We also included other working time-related covariates that may confound nurses' sickness absence: total number of working hours and total number of hours worked as bank (i.e., when voluntarily working shifts that cover temporary shortfalls in settings that are usually different to one's "home" role or ward). We additionally controlled for the number of previous sickness absence episodes, as well as part-time status, or working fewer than a median of 0.75 FTE (full time equivalent) hours per week in the previous quarter (i.e., median of ≤26 hours per week in the previous 13 weeks) [20]. Sickness episodes that were preceded by zero working hours in the previous month were removed. Following this predefined exclusion, all records and exposure windows were retained in models.

2.3 Statistical Methods, Sensitivity Analyses

We used random intercept logistic mixed models to estimate the association between shift pattern variables and the outcome of sickness absence. Repeated shift-level observations were clustered within nurses and accounted for by using nurse-level random intercepts. Intraclass correlation coefficients (ICC) were calculated to quantify clustering within nurses and wards. Significant clustering was observed at the nurse-level (adjusted ICC = 0.710) but not at the ward-level; therefore, additional ward-level effects were not included in models.

We tested the associations between shift pattern variables on nurses' sickness absence via: univariable models, which examined each main shift pattern variable independently, and full multivariable models, which included all shift pattern and control variables. Akaike information criterion (AIC) and Bayesian information criterion (BIC) values were used to compare alternative model specifications, including exposure window lengths and quadratic/cubic term forms, with lower values indicating improved model fit.

To test for multicollinearity between predictors, we calculated variance inflation factors (VIF), where values <5 indicated low multicollinearity [21]. Two sensitivity analyses were conducted: 1) testing variables with 28-day lookback windows to assess whether any observed effects persisted over a longer exposure period, and 2) re-analysing with pre-March 2020 data exclusively to compare and examine if associations were influenced by the onset of the COVID-19 pandemic in England. Shift variables were created with the *pandas* [22] and *datetime* python packages; modelling was undertaken using the *lme4* package in R (version 4.4.0) [23].

3. RESULTS

3.1. Descriptive Statistics

The final dataset contained 1,367,497 worked shifts and 19,876 sickness absence episodes from 7,515 registered nurses across 95 wards. The majority of worked shifts were from nurses working full-time (3789 nurses working 821,681 shifts (60%)) and sickness episodes lasted a median of 4 days long (IQR 2-8 days). The average number/proportion of each shift pattern configuration for full-time and part-time nurses for the entire study period is shown in

For peer review only - http://bmjopen.bmj.com/site/about/guidelines.xhtml

Table 1. Median shift length and the proportion of long shifts worked were similar between the groups, however full-time nurses worked more night shifts, quick returns, and shift rotations compared to part-time nurses. Part-time nurses had greater variability in their schedules, suggesting more heterogeneous work patterns.

Table 1. Shift patterns worked by full-time (FT) and part-time (PT) nurses in the previous 7 days

Shift Variables	FT Nurses		PT Nurses	
	Mean (SD)	Median	Mean (SD)	Median
**Total hours**	34.3 (5.0)	37.5	23.3 (9.5)	25.0
**N shifts**	2.9 (0.5)	3.0	2.0 (0.8)	2.0
**Average shift length**	12.0 (1.1)	12.5	11.7 (1.4)	12.5
**N long shifts**	2.5 (0.7)	3.0	1.7 (0.9)	2.0
**Proportion long shifts**	0.9 (0.2)	1.0	0.8 (0.3)	1.0
**N night shifts**	1.0 (0.8)	1.0	0.7 (0.6)	0.0
**Proportion night shifts**	0.3 (0.3)	0.3	0.3 (0.0)	0.0
**N long spells**	0.0 (0.0)	0.0	0.0 (0.0)	0.0
**N intense spells**	0.1 (0.1)	0.0	0.1 (0.1)	0.0
**N quick returns**	0.9 (0.4)	1.0	0.5(0.4)	0.0
**N short returns**	0.2 (0.2)	0.0	0.1(0.1)	0.0
**N shift rotations**	0.7 (0.4)	1.0	0.4 (0.4)	0.0

Supplementary table S1 presents a yearly snapshot of the shift pattern variables worked by full-time nurses specifically. Statistics demonstrate a stable pattern over the 5-year study period. Long (≥6 shifts) and intense (≥3 long or night shifts) consecutive spells were rare across the dataset, however, a slight increase in counts for the latter is noted from 2019 onwards. Similar increases are seen for the mean number of long shifts (2.4 in 2015 versus 2.5 in 2020), the mean proportion of night shifts (0.3 in 2015 versus 0.4 in 2020), and the number of quick returns (mean of 0.9 in 2015 versus 1.0 in 2020), indicating that these shift configurations became more frequent over the study period.

3.2. Multivariable Models

In the full multivariable model (Table 2), multicollinearity was low across all predictors (VIF < 5, excluding polynomial terms where elevated values were expected). Reduced odds of sickness absence were observed among nurses who had experienced a sickness episode within the preceding 7 days. In contrast, other covariates either had negligible or nonsignificant associations with sickness absence.

For every intense spell of work, quick return, and shift rotation, there was a 24%, 23% and 9% respective increase in the odds of sickness. Working long consecutive spells significantly and considerably decreased odds of sickness (OR 0.09, 95% CI 0.01-0.66), however this configuration was rare with only 1937 cases across the dataset.

Table 2. Shift pattern configurations worked in the previous 7 days and odds of sickness

Variables	Univariable Models		Multivariable Model	
	OR (95% CI)	Sig (P)	OR (95% CI)	Sig (P)
Main Shift Variables				

For peer review only - http://bmjopen.bmj.com/site/about/guidelines.xhtml

Proportion long shifts	0.80 (0.75-0.85)	<0.001	(refer to Figure 1)	<0.001
Proportion night shifts	1.06 (1.01-1.11)	0.014	(refer to Figure 1)	<0.001
N long spells	0.05 (0.01-0.38)	0.003	0.09 (0.01-0.66)	0.018
N intense spells	1.01 (0.96-1.06)	0.608	1.24 (1.16-1.32)	<0.001
N quick returns	0.97 (0.95-0.99)	0.006	1.23 (1.19-1.27)	<0.001
N short returns	0.94 (0.90-0.98)	0.007	1.05 (0.99-1.11)	0.111
N shift rotations	0.94 (0.92-0.96)	<0.001	1.09 (1.04-1.13)	<0.001
Covariates				
Total hours	0.99 (0.99-0.99)	<0.001	0.98 (0.97-0.98)	<0.001
Total bank hours	0.94 (0.94-0.94)	<0.001	0.94(0.94-0.95)	<0.001
N sickness episodes	0.57 (0.51-0.64)	<0.001	0.56 (0.50-0.63)	<0.001
Part-time status	1.07 (1.03-1.11)	<0.001	1.00 (0.96-1.04)	0.986
			**AIC**	145645
			**BIC**	145849

Furthermore, all variable terms for proportion of long shifts (linear, quadratic, cubic) and proportion of night shifts (linear, quadratic) returned as statistically significant (p≤0.001) (Table 3). Table 4 and Figure 1 show how odds of sickness fluctuated across proportions of long shifts and night shifts. Working ≥40% of shifts as long increased odds of sickness (relative to working no long shifts), with the highest odds observed around the 70% mark (OR 1.68, 95% CI 1.53-1.84). Working approximately 100% of shifts as night also slightly increased odds of sickness (relative to working no night shifts) (OR 1.07, 95% CI 1.01-1.13).

Table 3. Nonlinear terms for proportion of long shifts and night shifts in the previous 7 days

Variable	Term	β	SE	Sig (P)
Proportion long shifts	Linear	-2.09	0.44	<0.001
	Quadratic	8.73	1.12	<0.001
	Cubic	-6.69	0.70	<0.001
Proportion night shifts	Linear	-0.44	0.13	0.001
	Quadratic	0.50	0.14	<0.001

Table 4. Plotting values for nonlinear curves – proportion of long shifts and night shifts in the previous 7 days

	Prop long shifts		*Prop night shifts*	
**Proportion**	**Combined β terms**	**OR (95% CI)**	**Combined β terms**	**OR (95% CI)**
0	0.00	1.00 (1.00-1.00)	0.00	1.00 (1.00-1.00)
0.1	-0.13	0.88 (0.82-0.94)	-0.04	0.96 (0.94-0.99)
0.2	-0.12	0.88 (0.80-0.98)	-0.07	0.93 (0.90-0.98)
0.3	-0.02	0.98 (0.88-1.09)	-0.09	0.92 (0.87-0.97)
0.4	0.13	1.14 (1.03-1.26)	-0.09	0.91 (0.85-0.97)
0.5	0.30	1.35 (1.23-1.48)	-0.09	0.91 (0.85-0.98)
0.6	0.44	1.55 (1.42-1.70)	-0.08	0.92 (0.96-0.99)
0.7	0.52	1.68 (1.53-1.84)	-0.06	0.94 (0.89-1.00)
0.8	0.49	1.63 (1.48-1.79)	-0.03	0.97 (0.92-1.03)
0.9	0.31	1.36 (1.24-1.49)	0.01	1.01 (0.96-1.07)
1	-0.06	0.95 (0.87-1.03)	0.07	1.07 (1.01-1.13)

For peer review only - http://bmjopen.bmj.com/site/about/guidelines.xhtml

[**Insert Figure 1 here**]

As a sensitivity analysis, we re-estimated associations using a 28-day exposure window, the results of which are reported in Table S2, S3, and Figure S1. The 28-day multivariable model showed higher AIC and BIC values, indicating that the 7-day exposure window model provided a more parsimonious fit. For most shift pattern variables, the direction of effects was the same across the two exposure windows, although effect sizes were attenuated in the 28-day model. Both measures of consecutive working spells were not statistically significant in the 28-day model, indicating that the influence of this element of shift intensity was more pronounced over weekly timeframes. Further sensitivity analyses restricting data to pre-March 2020 records showed minimal differences in effect sizes (i.e., consistent direction and magnitude of effects with similar confidence intervals), confirming that our primary findings were not influenced by any unrealised, pandemic-related changes in working hours configurations.

4. DISCUSSION

The purpose of this observational study was to gain a comprehensive understanding of how adverse working time configurations are associated with sickness absence among registered nurses. This was achieved by analysing 1.4 million historical shift and sickness absence records collected from acute inpatient wards in two NHS hospital Trusts between 2015 and 2020. Compared to previous research on the relationships between shift type configurations and indicators of workforce wellbeing, this analysis explored the cumulative influence of patterns of work and rest, showing that higher proportions of long hours and night work, quick returns (having fewer than 11 hours of inter-shift recovery), intense spells of consecutive shifts (≥3 long or night shifts), and shift rotations were linked with increased odds of sickness absence.

In our study, nurses working high proportions of long shifts and night shifts were associated with increased odds of sickness absence, with the highest odds observed when over 70% of shifts were long (≥12 hours) or when working all night shifts in lookback windows. These findings mirror those of previous studies conducted within England. For example, in an analysis of 601,282 shift records from a large acute hospital, when 75% or more of shifts were worked as long shifts or night shifts in the past 7 days, the odds of sickness absence were increased when compared to working no long shifts (24% increase in odds) or day shifts only (12 % increase in odds) [11, 12]. Similarly, in a study that examined a pre-versus-post change in sickness absence rates in a large mental health hospital, an increase in the percentage of sickness hours per week (ranging from 0.73%-0.98%, or,1 shift per ward per week) was found following the organisational implementation of long shifts [15].

However, the nonlinear relationships observed in this study provide new and nuanced understanding of these associations. Although high proportions of long shifts were linked with increased odds of sickness (relative to working no long shifts), the magnitude of these odds diminished when proportions exceeded 70%. Furthermore, at the 100% mark, estimated odds fell below the null, however, confidence intervals indicated uncertainty around this estimate (OR 0.95, 95% CI 0.87–1.03). This pattern first appears counterintuitive given the demanding and fatiguing nature of long shifts, as evidenced by a substantial body of literature. One possible explanation for this observation is that nurses who work almost all

For peer review only - http://bmjopen.bmj.com/site/about/guidelines.xhtml

of their shifts as long shifts benefit from an element of consistency (i.e., working the same shift length), and therefore are able to establish routines and coping mechanisms accordingly. Rota consistency has been previously identified by nurses as a key scheduling practice that is supportive of their personal priorities in and outside of work [18], and the benefits of having some consistency may lessen the burden associated with long working hours. Self-selection similar to the 'healthy worker effect' [24] may also help explain this result, as individuals who can work demanding shifts and schedules continue to do so, while those more susceptible to sickness change their working patterns (or switch to standard schedules completely), thus skewing data and relationships toward the appearance of healthier outcomes.

Significant nonlinear relationships were also observed for the proportion of night shifts worked in the previous 7 days, where increased odds of sickness were only evident when working 100% night shifts (relative to working no night shifts). While the magnitude of this association was small (OR 1.07), small increases in individual sickness risk may still have operational relevance when concentrated night work occurs repeatedly across large nursing workforces. As an illustrative example, exposure to 100% night shifts was common in our study population, accounting for approximately 16% of all exposure windows (of which 2,845 were followed by sickness absence). This may correspond to roughly 186 additional sickness episodes, or 744 days lost to sickness absence based on the median episode duration of 4 days. Existing working time guidance advises against high exposure to night work, yet an oft-assumed benefit of this shift pattern is the avoidance of routine disruption that arises from rotating shiftwork. Our results contribute to the body of research that challenges this belief, as any benefits derived from consistency in this sense are likely overshadowed by the burden of night work in general (e.g., inability to reconcile work routines with personal/social routines, sustained metabolic dysregulation). In contrast, the reduced odds of sickness observed for proportions below 100% may be due to a reluctance to call in sick when scheduled to work night shifts (as part of a mixed day/night pattern) that arises from concerns about leaving wards understaffed or forfeiting pay premiums associated with working unsocial hours [25].

Another high demand shift configuration, intense spells of consecutive work (i.e., working more than 3 long shifts or night shifts consecutively) were associated with increased odds of sickness. This finding complements previous research on the implications of working many consecutive shifts: increased sickness absence rates when health care workers are working ≥ 4 or 5 consecutive night shifts, as well as consequences to other outcomes such as cognition, performance, occupational injury rates, and sleepiness when nurses are working consecutive long shifts [26-28]. However, results from qualitative research are mixed, with some nurses voicing their preference for consecutive shifts to enable longer periods of uninterrupted time off, while others call out the challenges of excessive consecutive shifts that impede one's ability to engage in life outside of work as a result of exhaustion or fatigue [18, 29].

The adverse associations observed when working consecutive shifts did not persist when examining long spells of continuous work (i.e., working more than 6 consecutive long or night shifts) as this variable significantly and considerably decreased odds of sickness in multivariable models. This configuration was rare across the dataset (<0.1%), which may indicate that when a long spell was worked, it was done so out of choice. Previous research suggests that choice and autonomy may influence the relationship between the demands of

For peer review only - http://bmjopen.bmj.com/site/about/guidelines.xhtml

shift work and staff wellness outcomes [30, 31], particularly for sickness absence as nurses may be less likely to call in sick for shift patterns they chose or requested. Other variables tested in our model that reflected some element of agency, such as the total number of bank hours and the number of sickness episodes in the lookback window, also significantly decreased odds of sickness. For this latter variable however, “agency” does not necessarily reflect making active choices over working time but rather could represent behavioural responses to absence monitoring policies enforced by employers. For example, some NHS Trusts make use of the controversial Bradford scoring tool [32], which penalises recurrent and short absences and thereby discourages employees from taking frequent sick leave, even when clinically necessary.

Lastly, shift configurations involving inadequate rest periods, namely quick returns and shift rotations, also demonstrated significantly increased odds of sickness. Of note, these findings were observed even though both variables were defined broadly to capture all relevant cases in the study population (i.e., including both day-to-night and night-to-day changes, defining quick returns as fewer than 11.5 hours of rest between shifts to account for the pervasive use of long shifts). Similar findings have been found in previous register-based sickness absence studies [13, 33], as well as in other research exploring outcomes such as stress, exhaustion, and fatigue [34]. Nevertheless, short returns – which represent a special case of inadequate rest (<48 hours between a night-to-day shift rotation) – did not return significant results. Having limited rest time between ending a night shift and starting a day shift has previously been identified as a problematic work/rest day configuration by nurses [18]; therefore, while this shift pattern may be linked with poorer wellbeing and work-life balance in some ways, it does not seem to associate with sickness as recorded through official mechanisms.

4.1. Limitations

The absence of demographic information in the underlying dataset (e.g., age, years in current role, active flexible working arrangements) restricted the ability to control for variables that may also impact relationships with sickness absence. However, these confounding effects were at least partially controlled for by accounting for outcome clustering at the individual/nurse level and other variables such as number of hours worked as ‘bank’ and the number of previous sickness episodes. Second, overtime work (working in excess of 37.5 hours per calendar week when averaged over a reference period) [35] is another shift variable previously shown to impact staff wellbeing, particularly when worked in excess of a working week with long and/or night shifts [1]. However, this variable could not be reliably assessed in the data, as elements of overtime work (e.g., extra hours worked at the end of a planned shift versus additional shifts that are worked in excess of the full-time limit) were recorded inconsistently. This limitation was in part mitigated by the inclusion of total working hours as a controlling variable, which also effectively captured extra work time that may not have been formally recorded as overtime.

5. CONCLUSIONS

This study of historical shift records provides detailed insight into the link between working hours and sickness absence among registered nurses working in acute adult hospital wards. Over the 5-year study period, changes in shift patterns were detected, with notable

For peer review only - http://bmjopen.bmj.com/site/about/guidelines.xhtml

increases in configurations such as spells of consecutive working days and inadequate inter-shift recovery. These configurations were also associated with higher odds of sickness, with the number of intense spells, quick returns, and shift rotations demonstrating effects in 7-day exposure windows. High proportions of long shifts and night shifts also significantly increased sickness absence, though these relationships were nonlinear and may have been influenced by other factors such as personal adaptability to adverse working conditions and discretion used when taking sick leave. Our findings highlight the organisational consequences of adverse working hours arrangements and can inform shift planning strategies that prioritise nurse wellbeing and proactive management of sickness absence.

FUNDING: The study dataset was derived from a project funded by the UK National Institute for Health and Care Research (NIHR) Health Services and Delivery Research Programme (Grant No. NIHR128056) and the NIHR Applied Research Collaboration (Wessex). The primary author was supported by the UKRI Economic and Social Research Council South Coast Doctoral Training Partnership (Grant No. ES/P000673/1). The views expressed are those of the author(s) and not necessarily those of the NIHR or the Department of Health and Social Care. These funders had no role in the design, conduct, or reporting of the study.

PATIENT AND PUBLIC INVOLVEMENT: Patients or the public were not involved in the design, conduct, reporting, or dissemination plans of this research. The study used de-identified electronic roster and sickness records of staff working in participating NHS hospital Trusts.

DECLARATION OF INTEREST: No competing interests to declare.

DATA AVAILABILITY: Due to the data sharing agreements with the providers, we are unable to share the source data.

REPORTING: This study is reported in accordance with the Strengthening the Reporting of Observational Studies in Epidemiology (STROBE) guidelines.

AUTHOR CONTRIBUTIONS: Talia Emmanuel (Guarantor): Conceptualisation, Formal analysis, Writing (original draft). **Peter Griffiths**: Conceptualisation, Methodology, Project administration, Formal analysis, Writing (original draft). **Carlos Lamas-Fernandez**: Formal analysis, Writing (original draft). **Chiara Dall'Ora**: Conceptualisation, Methodology, Project administration, Formal analysis, Writing (original draft).

For peer review only - http://bmjopen.bmj.com/site/about/guidelines.xhtml

REFERENCES

1. Health and Safety Executive (HSE). Managing shiftwork - Health and safety guidance. 2006.
2. Grzywacz JG. Shift Work and Its Implications for Everyday Work and Family Life: A Foundation and Summary. In: Iskra-Golec I, Barnes-Farrell J, Bohle P, editors. Social and Family Issues in Shift Work and Non Standard Working Hours. Cham: Springer International Publishing; 2016. p. 3–17.
3. Arlinghaus A, Bohle P, Iskra-Golec I, Jansen N, Jay S, Rotenberg L. Working Time Society consensus statements: Evidence-based effects of shift work and non-standard working hours on workers, family and community. Ind Health. 2019;57(2):184–200.
4. Moreno CRC, Marqueze EC, Sargent C, Wright Jr KP, Ferguson SA, Tucker P. Working Time Society consensus statements: Evidence-based effects of shift work on physical and mental health. Industrial Health. 2019;57(2):139–57.
5. Dall'Ora C, Ball J, Recio-Saucedo A, Griffiths P. Characteristics of shift work and their impact on employee performance and wellbeing: A literature review. International Journal of Nursing Studies. 2016;57:12–27.
6. Johnson CJ, Croghan E, Crawford J. The problem and management of sickness absence in the NHS: considerations for nurse managers. Journal of nursing management. 2003;11(5):336–42.
7. Dall'Ora C, Saville C, Rubbo B, Turner L, Jones J, Griffiths P. Nurse staffing levels and patient outcomes: A systematic review of longitudinal studies. International Journal of Nursing Studies. 2022;134:N.PAG–N.PAG.
8. Senek M, Robertson S, Ryan T, King R, Wood E, Tod A. The association between care left undone and temporary nursing staff ratios in acute settings: a cross-sectional survey of registered nurses. BMC Health Services Research. 2020;20(1):637.
9. NHS Digital. NHS Sickness Absence Rates 2024 [Available from: https://digital.nhs.uk/data-and-information/publications/statistical/nhs-sickness-absence-rates/april-2024.
10. NHS. NHS Staff Survey - National Results Briefing 2026 [updated March 2026. Available from: https://www.nhsstaffsurveys.com/results/national-results/.
11. Dall'Ora C, Ball J, Redfern O, Recio-Saucedo A, Maruotti A, Meredith P, et al. Are long nursing shifts on hospital wards associated with sickness absence? A longitudinal retrospective observational study. Journal of nursing management. 2019;27(1):19–26.
12. Dall'Ora C, Ball J, Redfern OC, Griffiths P. Night work for hospital nurses and sickness absence: a retrospective study using electronic rostering systems. Chronobiology international. 2020;37(9-10):1357–64.
13. Larsen AD, Ropponen A, Hansen J, Hansen ÅM, Kolstad HA, Koskinen A, et al. Working time characteristics and long-term sickness absence among Danish and Finnish nurses: A register-based study. International journal of nursing studies. 2020;112:103639.
14. Ropponen A, Koskinen A, Puttonen S, Härmä M. Exposure to working-hour characteristics and short sickness absence in hospital workers: A case-crossover study using objective data. International Journal of Nursing Studies. 2019;91:14–21.
15. Rodriguez Santana I, Anaya Montes M, Chalkley M, Jacobs R, Kowalski T, Suter J. The impact of extending nurse working hours on staff sickness absence: Evidence from a large mental health hospital in England. International journal of nursing studies. 2020;112:103611.
16. Griffiths P, Dall'Ora C, Saville C, Culliford D, Jones J, Meredith P, et al. Consequences, costs and cost-effectiveness of different workforce configurations in English acute hospitals: a longitudinal retrospective study using routinely collected data: National Institute for Health and Care Research; 2023 [Available from: https://fundingawards.nihr.ac.uk/award/NIHR128056.
17. Härmä M, Ropponen A, Hakola T, Koskinen A, Vanttola P, Puttonen S, et al. Developing register-based measures for assessment of working time patterns for

For peer review only - http://bmjopen.bmj.com/site/about/guidelines.xhtml

epidemiologic Studies. Scandinavian Journal of Work, Environment and Health. 2015;41(3):268–79.
18. Emmanuel T, Griffiths P, Lamas-Fernandez C, Ejebu OZ, Dall'Ora C. The important factors nurses consider when choosing shift patterns: A cross-sectional study. Journal of Clinical Nursing. 2024;33(3):998–1011.
19. Barker HR, Griffiths P, Dall'Ora C. "I don't think there's necessarily a one size fits all" negotiating competing priorities in nurse shift scheduling: a qualitative study. BMC nursing. 2025;24(1):1048.
20. Van Bastelaer A, Lemaître G, Marianna P. The definition of part-time work for the purpose of international comparisons. OECD Labour Market and Social Policy Occasional Papers. 1997(22).
21. James G, Witten D, Hastie T, Tibshirani R. An Introduction to Statistical Learning: with Applications in R: Springer New York; 2013.
22. McKinney W. Python for Data Analysis: Data Wrangling with pandas, NumPy, and Jupyter: O'Reilly Media; 2022.
23. Bates D, Mächler M, Bolker B, Walker S. Fitting Linear Mixed-Effects Models Using lme4. Journal of Statistical Software. 2015;67(1):1 – 48.
24. Ritonja J, Aronson KJ, Matthews RW, Boivin DB, Kantermann T. Working Time Society consensus statements: Individual differences in shift work tolerance and recommendations for research and practice. Industrial health. 2019;57(2):201–12.
25. NHS Employers. Unsocial hours payments 2022 [Available from: https://www.nhsemployers.org/articles/unsocial-hours-payments.
26. Hopcia K, Dennerlein JT, Hashimoto D, Orechia T, Sorensen G. Occupational injuries for consecutive and cumulative shifts among hospital registered nurses and patient care associates: a case-control study. Workplace health & safety. 2012;60(10):437–44.
27. Hirsch Allen AJ, Park JE, Adhami N, Sirounis D, Tholin H, Dodek P, et al. Impact of work schedules on sleep duration of critical care nurses. American journal of critical care : an official publication, American Association of Critical-Care Nurses. 2014;23(4):290–5.
28. James L, Elkins-Brown N, Wilson M, James SM, Dotson E, Edwards CD, et al. The effects of three consecutive 12-hour shifts on cognition, sleepiness, and domains of nursing performance in day and night shift nurses: A quasi-experimental study. International journal of nursing studies. 2021;123:104041.
29. Ejebu OZ, Dall'Ora C, Griffiths P. Nurses' experiences and preferences around shift patterns: A scoping review. PLoS ONE. 2021;16(8 August).
30. Demerouti E, Bakker AB, Nachreiner F, Schaufeli WB. The job demands-resources model of burnout. Journal of Applied psychology. 2001;86(3):499.
31. Dall'Ora C, Ejebu OZ, Ball J, Griffiths P. Shift work characteristics and burnout among nurses: cross-sectional survey. Occupational Medicine. 2023;73(4):199–204.
32. Taylor S. People Resourcing: Managing Absence. 3rd ed: Chartered Institute of Personnel And Development; 2005.
33. Rosenström T, Härmä M, Kivimäki M, Ervasti J, Virtanen M, Hakola T, et al. Patterns of working hour characteristics and risk of sickness absence among shift-working hospital employees: A data-mining cohort study. Scandinavian Journal of Work, Environment and Health. 2021;47(5):395–403.
34. Min A, Min H, Hong HC. Work schedule characteristics and fatigue among rotating shift nurses in hospital setting: An integrative review. Journal of nursing management. 2019;27(5):884–95.
35. NHS Employers. NHS Terms and Conditions of Service Handbook 2024 [Available from: https://www.nhsemployers.org/publications/tchandbook.

For peer review only - http://bmjopen.bmj.com/site/about/guidelines.xhtml

Figure 1. Cubic and quadratic curves for proportion of long shifts and night shifts in the previous 7 days

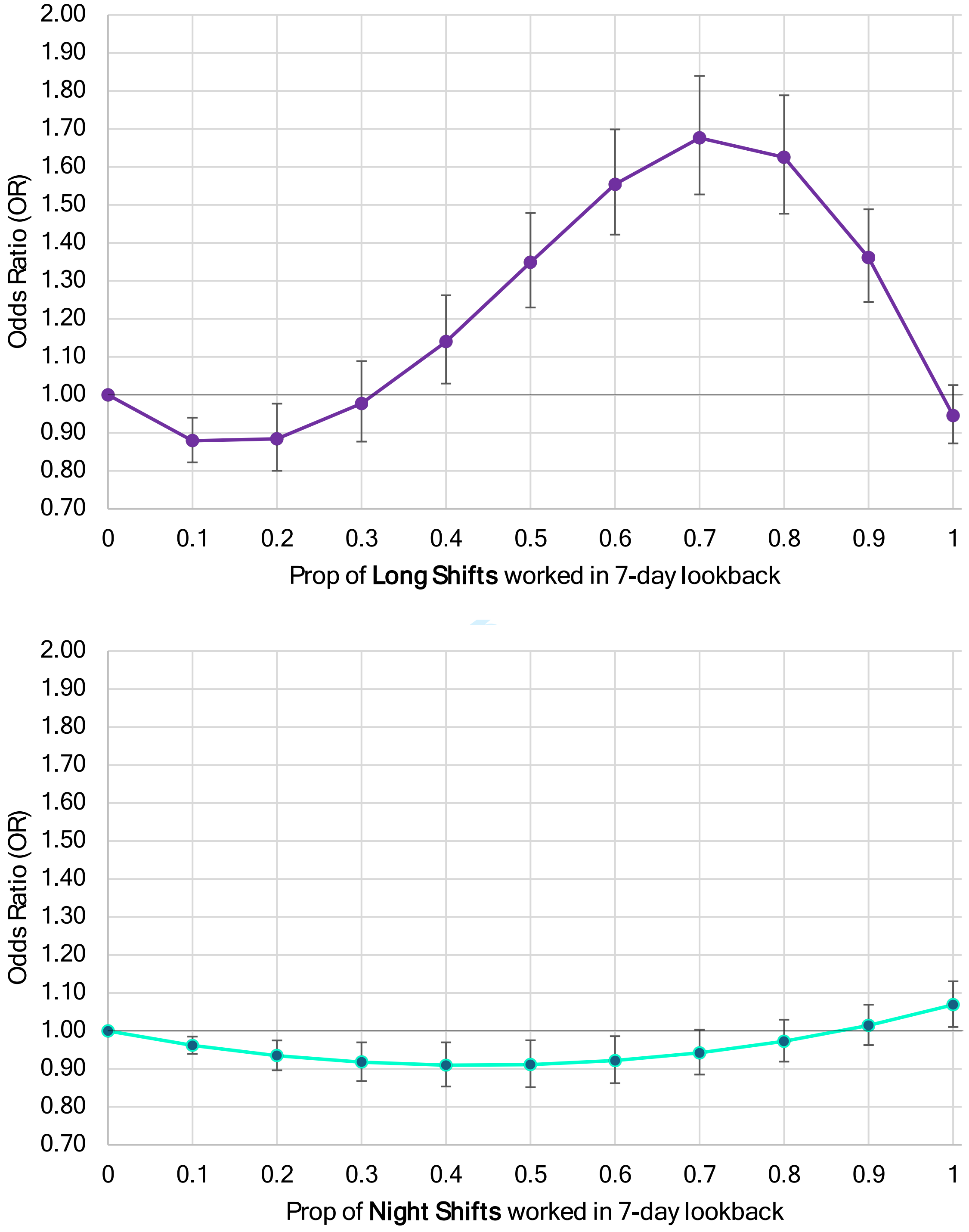

SUPPLEMENTARY MATERIAL

Supplementary Table S1. Shift pattern configurations worked by full-time nurses in the previous 7 days

Shift Variables	Overall		2015		2016		2017		2018		2019		2020	
	Mean (SD)	Median	Mean (SD)	Median	Mean (SD)	Median	Mean (SD)	Median	Mean (SD)	Median	Mean (SD)	Median	Mean (SD)	Median
Total hours	34.3 (5.0)	37.5	33.9 (4.8)	37.5	33.9 (4.7)	37.5	34.1 (4.5)	37.5	33.9 (4.9)	37.5	34.6 (5.3)	37.5	35.0 (5.6)	37.5
N shifts	2.9 (0.5)	3.0	2.9 (0.5)	3.0	2.9 (0.5)	3.0	2.9 (0.4)	3.0	2.9 (0.5)	3.0	2.9 (0.5)	3.0	2.9 (0.5)	3.0
Avg shift length	12.0 (1.1)	12.5	12.0 (1.1)	12.5	12.0 (1.1)	12.5	12.0 (1.1)	12.5	12.0 (1.1)	12.5	12.0 (1.0)	12.5	12.1 (1.0)	12.5
N long shifts	2.5 (0.7)	3.0	2.4 (0.8)	3.0	2.4 (0.7)	3.0	2.5 (0.7)	3.0	2.4 (0.7)	3.0	2.5 (0.8)	3.0	2.5 (0.8)	3.0
Prop long shifts	0.9 (0.2)	1.0	0.9 (0.3)	1.0	0.9 (0.2)	1.0	0.9 (0.2)	1.0	0.9 (0.2)	1.0	0.9 (0.2)	1.0	0.9 (0.2)	1.0
N night shifts	1.0 (0.8)	1.0	1.0 (0.7)	1.0	1.0 (0.7)	1.0	1.0 (0.7)	1.0	1.0 (0.8)	1.0	1.0 (0.8)	1.0	1.1 (0.8)	1.0
Prop night shifts	0.3 (0.3)	0.3	0.3 (0.2)	0.3	0.3 (0.2)	0.3	0.3 (0.2)	0.3	0.3 (0.3)	0.3	0.4 (0.3)	0.3	0.4 (0.3)	0.3
N long spells	0.0 (0.0)	0.0	0.0 (0.0)	0.0	0.0 (0.0)	0.0	0.0 (0.0)	0.0	0.0 (0.0)	0.0	0.0 (0.0)	0.0	0.0 (0.0)	0.0
N intense spells	0.1 (0.1)	0.0	0.1 (0.1)	0.0	0.1 (0.1)	0.0	0.1 (0.1)	0.0	0.1 (0.1)	0.0	0.2 (0.2)	0.0	0.2 (0.2)	0.0
N quick returns	0.9 (0.4)	1.0	0.9 (0.4)	1.0	0.9 (0.4)	1.0	0.9 (0.4)	1.0	0.9 (0.4)	1.0	1.0 (0.4)	1.0	1.0 (0.5)	1.0
N short returns	0.2 (0.2)	0.0	0.2 (0.2)	0.0	0.2 (0.2)	0.0	0.2 (0.2)	0.0	0.2 (0.2)	0.0	0.2 (0.2)	0.0	0.2 (0.2)	0.0
N shift rotations	0.7 (0.4)	1.0	0.7 (0.4)	1.0	0.7 (0.4)	1.0	0.7 (0.4)	1.0	0.7 (0.4)	1.0	0.7 (0.4)	1.0	0.7 (0.5)	1.0

For peer review only

Supplementary Table S2. Shift pattern configurations worked in the previous 28 days and odds of sickness

Variables	Univariable Models		Multivariable Model	
	OR (95% CI)	Sig (P)	OR (95% CI)	Sig (P)
Main Shift Variables				
Prop long shifts	1.11 (1.02-1.20)	0.013	(refer to Figure S1)	<0.001
Prop night shifts	0.98 (0.91-1.05)	0.610	(refer to Figure S1)	<0.001
N long spells	0.69 (0.59-0.81)	<0.001	0.98 (0.83-1.16)	0.859
N intense spells	0.95 (0.93-0.97)	<0.001	0.99 (0.96-1.02)	0.482
N quick returns	0.98 (0.97-0.99)	<0.001	1.04 (1.02-1.05)	<0.001
N short returns	0.96 (0.94-0.98)	<0.001	0.97 (0.94-1.00)	0.030
N shift rotations	1.00 (0.99-1.01)	0.929	1.04 (1.02-1.06)	<0.001
Covariates				
Mean weekly hours	0.99 (0.99-0.99)	<0.001	0.99 (0.99-0.99)	<0.001
Total bank hours	0.98 (0.98-0.98)	<0.001	0.98 (0.98-0.98)	<0.001
N sickness episodes	0.68 (0.65-0.71)	<0.001	0.64 (0.61-0.68)	<0.001
Part-time status	1.07 (1.03-1.11)	<0.001	0.99 (0.95-1.03)	0.635

Supplementary Table S3. Nonlinear terms for proportion of long shifts and night shifts in the previous 28 days

Variable	Term	Beta	Sig (P)
Prop long shifts	Linear	-1.636	<0.001
	Quadratic	6.5484	<0.001
	Cubic	-4.748	<0.001
Prop night shifts	Linear	-0.484	<0.001
	Quadratic	0.546	0.001

For peer review only

Supplementary Figure S1. Cubic and quadratic curves for proportion of long shifts and night shifts in the previous 28 days

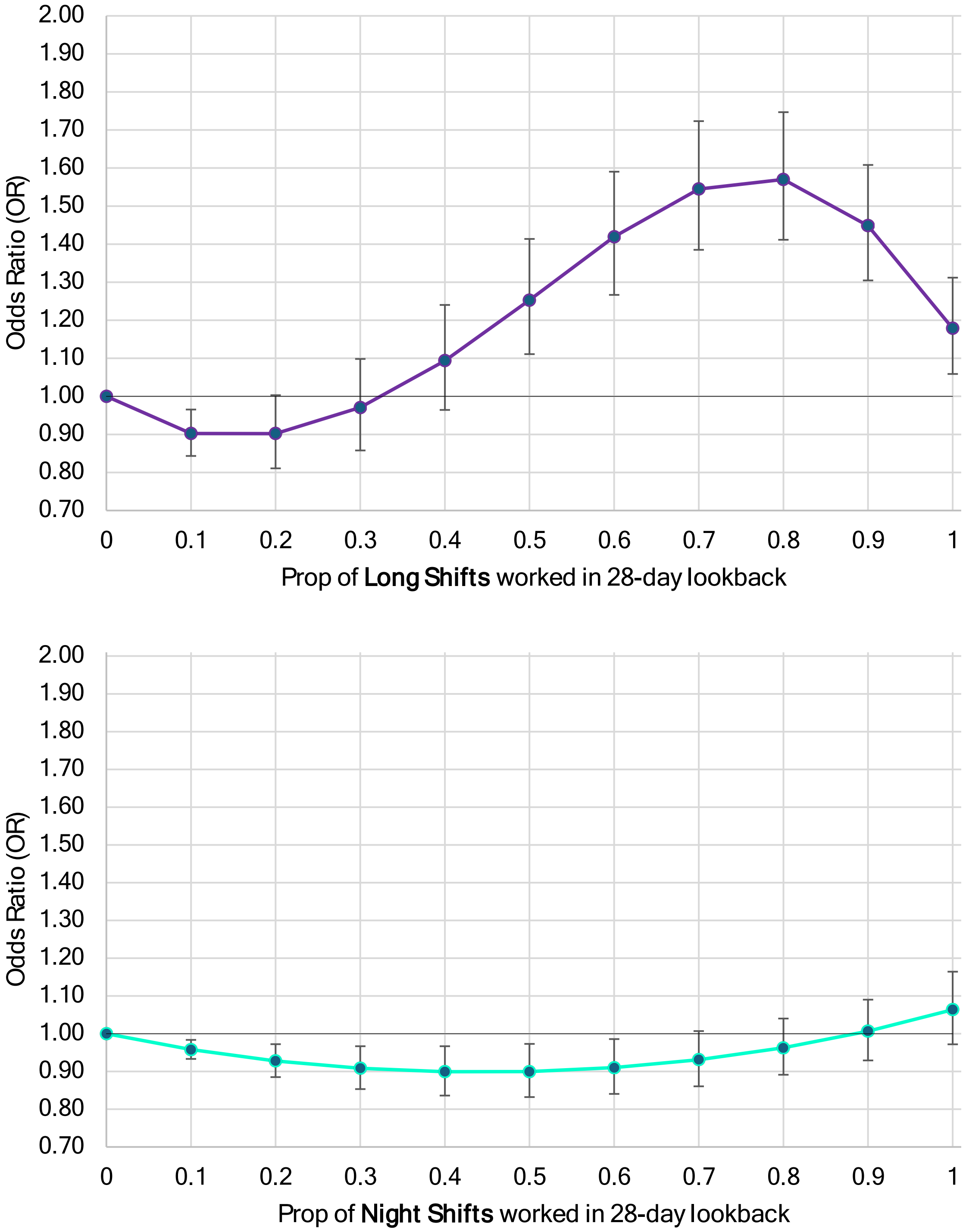

Supplement: Author's manuscript [file bmjopen-2026-120066.draft_revisions.pdf]
